# Structural basis for RNA translocation by DEAH-box ATPases

**DOI:** 10.1093/nar/gkz150

**Published:** 2019-03-04

**Authors:** Florian Hamann, Marieke Enders, Ralf Ficner

**Affiliations:** Department of Molecular Structural Biology, Institute for Microbiology and Genetics, GZMB, Georg-August-University Goettingen, Justus-von-Liebig-Weg 11, 37077 Goettingen, Germany

## Abstract

DEAH-box adenosine triphosphatases (ATPases) play a crucial role in the spliceosome-mediated excision of pre-mRNA introns. Recent spliceosomal cryo-EM structures suggest that these proteins utilize translocation to apply forces on ssRNAs rather than direct RNA duplex unwinding to ensure global rearrangements. By solving the crystal structure of Prp22 in different adenosine nucleotide-free states, we identified two missing conformational snapshots of genuine DEAH-box ATPases that help to unravel the molecular mechanism of translocation for this protein family. The intrinsic mobility of the RecA2 domain in the absence of adenosine di- or triphosphate (ADP/ATP) and RNA enables DEAH-box ATPases to adopt different open conformations of the helicase core. The presence of RNA suppresses this mobility and stabilizes one defined open conformation when no adenosine nucleotide is bound. A comparison of this novel conformation with the ATP-bound state of Prp43 reveals that these ATPases cycle between closed and open conformations of the helicase core, which accommodate either a four- or five-nucleotide stack in the RNA-binding tunnel, respectively. The continuous repetition of these states enables these proteins to translocate in 3′-5′ direction along an ssRNA with a step-size of one RNA nucleotide per hydrolyzed ATP. This ATP-driven motor function is maintained by a serine in the conserved motif V that senses the catalytic state and accordingly positions the RecA2 domain.

## INTRODUCTION

In eukaryotes, the removal of non-coding intron sequences in pre-mRNAs is performed by the spliceosome, a multimegadalton molecular machine composed of numerous RNA and protein components (1–3). The spliceosome is a highly dynamic complex in terms of conformation and composition. All components are sequentially assembled in a stepwise manner and only a few of them remain constantly part of the complex. In order to remove one intron, the complex has first to be assembled mainly by the recruitment of five small nuclear ribonucleoprotein particles, named U1, U2, U4/U6 and U5, until reaching a preformed but catalytically inactive complex. After catalytic activation, two transesterification reactions ensure the excision of the intron and the ligation of the exons. Once this task is completed, the complex is completely disassembled and each component becomes available for another round of splicing.

The key triggering components of the spliceosome driving the transition from state to state are a set of so-called RNA helicases (4–6). The first assembly steps are governed by the DEAD-box helicases and the activated spliceosome is achieved by the action of the Ski2-like helicase Brr2. The catalytic activation, the catalysis itself as well as the final disassembly steps are driven by so-called DEAH-box helicases. They all belong to the helicase superfamily 2 (SF2) and share at least eight conserved sequence motifs (motifs I, Ia, Ib, II, III, IV, V and VI) spread over two RecA-like domains that together form the helicase core (7). These motifs are vital for the function of these proteins as they facilitate ATP and RNA binding, ATP hydrolysis as well as coupling ATP hydrolysis to RNA unwinding/translocation (8–11). Ski2-like and DEAH-box ATPases additionally possess auxiliary domains positioned C-terminally to the helicase core. Together they form a tunnel for single-stranded nucleic acid binding (12–14).

The release of the mature messenger RNA (mRNA) after intron cleavage is performed by the DEAH-box ATPase Prp22 (15,16). It has multiple additional functions such as proof-reading, suppression of splicing of aberrant intermediates with suboptimal 3′ splice site and it participates in the second splicing reaction (17–19). Functionally Prp22 shares features common for other DEAH-box ATPases. They have the common ability to hydrolyze any kind of nucleoside triphosphates (NTPs) and the ATPase activity is stimulated by the presence of single-stranded RNA (ssRNA) (11,20). They have no requirements for a specific RNA nucleotide sequence and generally unwind RNA duplexes with a 3′ overhang and with a 3′-5′ polarity (20,21).

DEAH-box ATPases have long been assigned as the key players responsible for enabling the sequential transition of the last spliceosomal stages. The primary function they have been attributed in the spliceosome is to induce conformational and compositional rearrangements of the different complexes by direct unwinding of RNA duplexes and have been termed helicases (16,22–24). While most of them indeed show *in vitro* helicase activity, none of the recent cryo-EM structures containing one of these so-called helicases shows an RNA duplex that could serve as a direct target (25–28). Instead, single-stranded RNA segments are usually spotted close by. The interaction of these proteins with spliceosomal RNA components is vital for their function and as direct duplex unwinding seems unlikely, the ATPases have to fulfill their tasks by other means. Disruption of duplexes in most SF2 members is tightly linked to processivity and while the spliceosomal context might not require DEAH-box members to be actual unwindases, translocation along a single-stranded RNA might still be needed. Due to their attachment to other protein factors at the spliceosomal peripheries, they are likely not mobile and do not translocate on long loose RNA stretches. Thus, translocation might enable the ATPases to apply forces on the RNA, a mechanism recently described as winching (19). This could not only ensure rearrangements in the vicinity of the ATPase, but these mechanical forces might as well be transmitted to more interior parts of the spliceosome potentially leading to indirect duplex disruptions. Thus, the sole translocation could be the primary function of DEAH-box ATPases in the spliceosome and a better understanding of the molecular mechanism of translocation might help to understand larger dynamic events of the spliceosome.

Although biochemical and genetic studies over the past decades and more recent structural cryo-EM studies have helped to locate and characterize DEAH-box ATPases in the context of the spliceosome, detailed mechanistic insights of how they function as a molecular machine are still scarce (11,21,25–30). Crystallographic approaches, mainly on Prp43, describing different catalytic states have started to deepen the understanding of how this protein family might work (13,14,23,31,32). A key feature of these ATPases seems to be their conformational rearrangements that are strongly linked to their catalytic state. Thus, having structural snapshots of all possible catalytic states will help to fully explain their underlying molecular mechanism. Here, we present the first structures of the functional core of the spliceosomal DEAH-box ATPase Prp22 from *Chaetomium thermophilum* in two catalytic states previously undescribed for genuine DEAH-box ATPases that contribute to the completion of the catalytic state repertory. A Prp22 Apo structure highlights the mobility of the RecA2 domain in the absence of any adenosine nucleotide. A complex structure of Prp22 with a poly-U_9_ ssRNA in the absence of ADP/ATP suggests that the RecA2 domain is stabilized by the interaction with the sugar-phosphate backbone of the RNA via a conserved and adenosine nucleotide-independent interaction pattern. The absence of ATP/ADP allows the helicase core to adopt an open conformation accommodating a one nucleotide longer RNA stretch in the binding tunnel. We identified a serine in motif V playing a crucial role in sensing the catalytic state and thereby regulating the conformational dynamics of the helicase core. Comparing these new catalytic snapshots with known ones from Prp43, we propose a molecular model of the translocation mechanism of genuine DEAH-box ATPases based on a step-size of one RNA nucleotide per hydrolyzed ATP.

## MATERIALS AND METHODS

### Protein production

Prp22 from *C. thermophilum* (ctPrp22) was identified as an homolog of *Saccharomyces cerevisiae* Prp22 (scPrp22) using the NCBI BLAST search tool (GenBank: EGS21698.1, 33). An N-terminally truncated version missing the first 545 residues was amplified by polymerase chain reaction from a total DNA preparation. The amplified DNA product was cloned into pGEX-6p1 vector using the EcoRI and NotI restriction sites. The forward primer contained the sequence for the Tobacco Etch Virus (TEV) cleavage site directly prior to the ctPrp22 construct. The GST fusion protein was recombinantly expressed in Rosetta 2 (DE3) cells using an autoinduction protocol (34). Harvested cells were disrupted using a microfluidizer (Microfluidics) in 50 mM Tris/HCl (pH 7.5), 500 mM NaCl, 5% (v/v) glycerol and 10 mM ethylenediaminetetraacetic acid (EDTA). The lysate was clarified via ultracentrifugation at 30 000 g and 4°C for 30 min. The clarified lysate was loaded on a Glutathione Sepharose column (GE Healthcare) at 20°C. Potentially bound nucleic acids were removed using a wash step with lysate buffer supplemented with 2 M LiCl and the protein was subsequently eluted with 30 mM reduced glutathione. The GST-tag was proteolytically cleaved with 1:50 (w/w) TEV protease overnight at 4°C. Remaining impurities and the tag were removed using a Superdex 75 gel-filtration column coupled to a Glutathione Sepharose column in 20 mM Tris/HCl (pH 7.5), 200 mM NaCl, 5% glycerol and 2 mM MgCl_2_. The protein was concentrated to 7 mg ml^−1^ with an Amicon Ultra centrifugal concentrator (Merck). About 200 μl protein samples were flash frozen in liquid nitrogen and stored at −80°C. ctPrp43, ctPrp43-S387A, ctPrp43-S387G, ctPrp22-S837A, ctPrp22-S837G and ctPrp22-S837P were expressed and purified using the same protocol as described for ctPrp22.

### Crystallization

ctPrp22 and ctPrp22-S837A were diluted to 2.5 mg ml^−1^ (32.76 μM) and incubated with a 10-fold molar excess of ADP, a 20-fold molar excess of BeSO_4_, a 60-fold molar excess of NaF and a 2.5-fold molar excess of U_12_-RNA (AXOlabs, Germany) for at least 30 min at 4°C. Crystallization was performed via the sitting-drop vapor-diffusion technique by mixing 1 μl complex solution with 1 μl crystallization buffer. ctPrp22 Apo crystals were obtained in 100 mM Bis-Tris propane (pH 6.5), 400 mM K-Thiocyanate and 20% (w/v) PEG3350 and ctPrp22+RNA and ctPrp22-S837A+RNA crystals in 100 mM Bis-Tris propane (pH 8.5), 250 mM NaF and 22% (w/v) PEG3350. All crystals were obtained after 2–3 days at 20°C. ctPrp43-S837A and ctPrp43-S387G crystals were obtained as described in Tauchert *et al.* (14).

### Data collection and processing

Crystals of ctPrp22 and ctPrp22-S837A were cryoprotected in the respective reservoir solution complemented with 10% (w/v) PEG400 and 5% (w/v) glycerol and flash-cooled in liquid nitrogen prior to data collection. Crystals of ctPrp43-S387A and ctPrp43-S387G were not additionally cryoprotected prior to flash-cooling. X-ray diffraction data were collected at 100 K on beamline P13 (ctPrp22) and beamline P14 (ctPrp22-S837A and ctPrp43-S387A), PETRA III, DESY (Hamburg, Germany), as well as on Rigaku MicroMax-003 equipped with a Dectris Pilatus 2K detector (ctPrp43-S387G). The *XDS* package was used for data processing. The highest resolution limit was estimated using a minimum I/σ(I) of 1.5 and a minimum CC_1/2_ of 60% as cutting criteria (35).

### Structure solution, refinement and analysis

The ctPrp22+RNA complex structure was solved by molecular replacement using *Phaser* with the structure of ctPrp2 (PDB ID: 6fa5) used as a search model (36,37). Prior to molecular replacement, the amino acid sequence of the ctPrp2 model was modified to match the sequence of ctPrp22 using the *chainsaw* utility in the *CCP4* suite (38). Due to the different conformations of ctPrp22 in the presented structures, no molecular replacement solution could be obtained with this preliminary model. After splitting it into three domains (RecA1, RecA2 and C-terminal) and using them as subsequent search ensembles for molecular replacement, a solution was found. The structure of ctPrp22-Apo was solved using the same individual domains as search ensembles but from the final model of the ctPrp22+RNA complex. ctPrp22 Apo contains two molecules and ctPrp22+RNA four molecules per asymmetric unit. The models were manually built with *Coot* and refinements were performed with *Refmac5* (39,40). Both structures were refined using the RecA1, RecA2 and C-terminal domains as translation, libration and screw-axis (TLS) groups and with the inclusion of automatically generated local non-crystallographic symmetry (NCS) restraints (41). The ctPrp22-Apo structure was additionally refined with external restraints using the ctPrp22 model from the RNA complex as a reference structure (42). Serine mutant structures of ctPrp22 and ctPrp43 were solved using the corresponding wild-type structure (ctPrp22-S837A: 6i3p; ctPrp43-S387A and ctPrp43-S387G: 5ltj) for molecular replacement. The quality of the final model was assessed using *MolProbity* (43). Structure superpositions were calculated with *LSQMAN* (44). Figures were prepared with *PyMOL* (v.1.8, Schrödinger) and *Chimera* (45).

### ATPase activity assay

The ATPase activity measurements of ctPrp22 and ctPrp43 were conducted with a nicotinamide adenine dinucleotide (NADH) dependent coupled enzymatic assay (46). The decrease of the NADH absorption at 340 nm as a direct effect of the ATP consumption was recorded over time with a VICTOR Nivo Multimode Microplate Reader (PerkinElmer). All reactions were performed in triplicates of 150 μl each at 25°C in 25 mM Bis-Tris propane (pH 7.5), 150 mM KCl and 3 mM MgCl_2_ supplemented with 250 nM NADH, 500 nM phosphoenolpyruvate, 6–8.3 U ml^−1^ pyruvate kinase and 9–14 U ml^−1^ lactic dehydrogenase. The ATP concentration was varied from 0 to 2 mM. Measurements in the presence of RNA were conducted with a 20-fold molar excess over ctPrp22 or ctPrp43 of a A_20_-ssRNA (AXOlabs). ctPrp22 was used at a concentration of 0.4 μM and ctPrp43 at 5 μM. *K*_m_ and *k*_cat_ were calculated by fitting the experimental data according to the Michaelis and Menten equation using QtiPlot (v.0.9.8.9).

### RNA binding assay

The RNA binding in dependence of different adenosine nucleotides was performed via fluorescence polarization measurements using a VICTOR Nivo Multimode Microplate Reader (PerkinElmer). The binding of 6 nM 3′ 6-carboxyfluorescein-labeled A_20_-RNA to up to 70 μM ctPrp22 and 120 μM ctPrp43 was monitored in 20 mM Tris/HCl (pH 7.5), 200 mM NaCl, 5% glycerol and 3 mM MgCl_2_. Measurements in presence of ADP/adenylyl imidodiphosphate (AMPPNP) were performed with a 50-fold molar excess of the nucleotide over ctPrp22 or ctPrp43, except for ADP-dependent measurements of ctPrp22 where a constant concentration of 3.5 mM of the nucleotide was used throughout all measurements. All reactions were performed in 50 μl and measured as a set of triplicates. For all measurements an excitation wavelength of 480 nm was set and the emission was detected at 530 nm for 500 ms. The measured fluorescence polarization was normalized and fitted according to Rossi and Taylor (47) using QtiPlot (v.0.9.8.9).

### Circular dichroism spectroscopy

In order to verify the structural integrity of ctPrp22-S837P, far-UV spectra (185–260 nm) of this mutant protein and the wild-type protein were recorded using a Chirascan CD spectrometer (Applied Photophysics). Measurements were performed in 20 mM Na-phosphate buffer at 20°C with a protein concentration of 0.1 mg ml^−1^. For each construct, 15 spectra of 1 s accumulation were averaged for the final spectrum. The CD data are presented as mean residue ellipticity (θ) and fitted using QtiPlot (v.0.9.8.9) (48).

## RESULTS AND DISCUSSION

### Prp22 architecture and function

The crystallographic structure determination of the spliceosomal DEAH-box ATPase Prp22 presented here was performed with the protein from the ascomycete *C. thermophilum* (ctPrp22). Previous studies on other closely related spliceosomal DEAH-box members, Prp2 and Prp43, have demonstrated that proteins from this organism are suited for crystallographic investigations as they might have an increased tendency to crystallize when compared with orthologs from *S. cerevisiae* or *Homo sapiens* (14,32,37,49). ctPrp22 exhibits an amino acid sequence identity of 54.7 and 57.7% to yeast and human Prp22, respectively. As the N-terminal region of Prp22 is predicted to be in part unfolded and recent structural studies on Prp2 and Prp43 have proven that the helicase core and the C-terminal domains are the key domains involved in the function of this ATPase family, an N-terminally truncated version of ctPrp22 lacking the first 545 amino acids was used for structure determination (Supplementary Figure S1A). The construct lacking the N-terminus has a sequence identity of 65.2 and 73.5% to yeast and human Prp22, respectively. Two different crystal structures of ctPrp22 were obtained, one with a bound single-stranded RNA and another one with no interaction partner (Table 1).

**Table 1.** Data collection and refinement statistics

Data collection	ctPrp22 Apo	ctPrp22 + RNA	ctPrp22-S837A + RNA	ctPrp43-S387A + ADP-BeF_3_^−^	ctPrp43-S387G + ADP-BeF_3_^−^
Space group	P2_1_2_1_2_1_	P2_1_2_1_2_1_	P2_1_2_1_2_1_	P2_1_2_1_2_1_	P2_1_2_1_2_1_
a (Å)	67.5	139.8	139.6	88.8	88.6
b (Å)	93.6	140.5	140.0	105.5	103.0
c (Å)	218.4	159.6	164.7	119.2	118.9
X-ray source	P13, PETRA III, DESY	P13, PETRA III, DESY	P14, PETRA III, DESY	Rigaku, MicroMax-003	P14, PETRA III, DESY
Resolution range (Å)	86.06–3.25	49.24–2.75	106.49–2.70	48.22–2.20	77.85–2.70
	(3.35–3.25)	(2.87–2.75)	(2.82–2.70)	(2.30–2.20)	(2.80–2.70)
No. of unique reflections	22 460	82 130	88 822	57 004	30 459
Completeness (%)	99.5 (96.3)	99.9 (100.0)	99.7 (99.8)	99.1 (93.0)	99.8 (99.8)
*R*_merge_ (%)	11.3 (106.0)	11.0 (146.9)	7.7 (147.4)	6.6 (56.6)	11.3 (147.4)
Average I/σ(I)	12.04 (1.52)	14.28 (1.51)	19.56 (1.69)	20.81 (2.51)	14.78 (1.64)
Redundancy	5.20 (4.98)	7.79 (8.15)	9.25 (9.44)	7.47 (4.20)	9.11 (9.24)
CC_1/2_	99.8 (65.7)	99.9 (75.0)	100.0 (70.5)	99.9 (81.9)	99.9 (64.8)
Wilson B (Å^2^)	76.35	72.84	81.23	36.44	71.31
**Refinement**					
Resolution (Å)	86.06–3.25	49.24–2.75	106.49–2.70	35.61–2.20	77.85–2.70
	(3.36–3.25)	(2.82–2.75)	(2.77–2.70)	(2.24–2.20)	(2.79–2.70)
No. of reflections	21 337	78 023	84 380	56 985	30 443
*R*_work_ (%)	24.65 (36.6)	22.21 (37.6)	21.22 (37.6)	18.36 (27.0)	22.86 (42.9)
*R*_free_ (%)	26.06 (40.1)	25.23 (39.8)	26.15 (38.6)	20.81 (28.3)	25.37 (46.9)
Molecules per asymmetric unit	2	4	4	1	1
Total number of atoms	9208	20 540	20 594	6472	5741
Protein residues	1166	2509	2510	703	703
Water molecules	/	/	1	704	39
Nucleic acid molecules	/	4	4	/	/
r.m.s. deviations					
Bond length (Å)	0.012	0.011	0.009	0.009	0.010
Bond angles (°)	1.74	1.56	1.51	0.91	0.97
Mean B-factors (Å^2^)					
Protein	112.34	78.48	93.32	34.99	77.09
RNA	/	81.23	101.30	/	/
Ramachandran statistics					
Favored (%)	95.01	97.03	96.55	97.72	98.00
Allowed (%)	4.90	2.81	3.45	2.28	2.00
Outliers (%)	0.09	0.16	0.00	0.00	0.00
PDB ID	6i3o	6i3p	6qic	6qid	6qie

**Figure 1.**
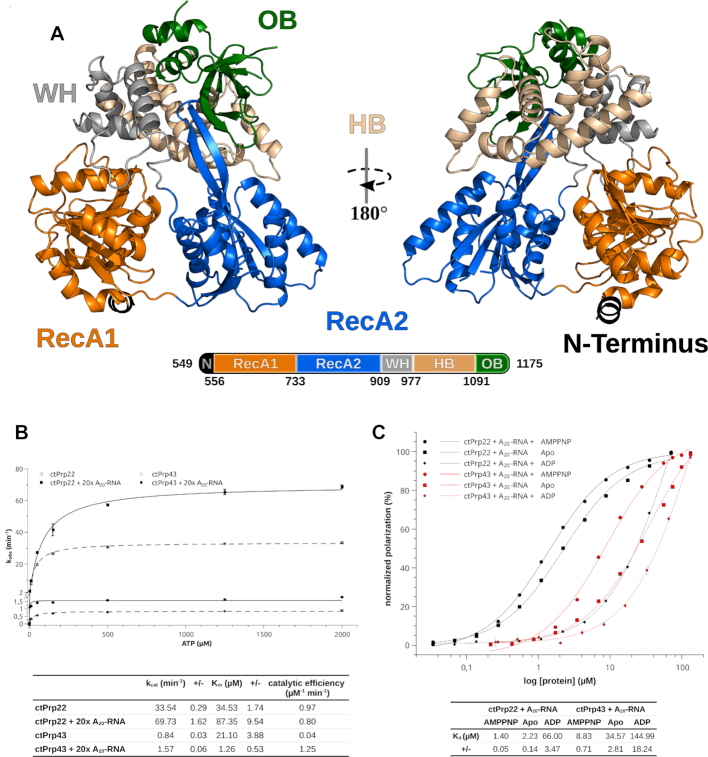
Structural overview of Prp22 from *Chaetomium thermophilum* and functional comparison of ctPrp22 and ctPrp43. (**A**) Structural overview of Prp22 from *C. thermophilum*. The model of ctPrp22 is displayed as a cartoon representation. Residues from the truncated N-terminus (549–556) are shown in black, the RecA1 domain (557–733) in orange, the RecA2 domain (734–909) in blue, the WH domain (WH; 910–977) in gray, the HB domain (HB; 978–1091) in wheat and the OB fold (OB; 1092–1175) in green. (**B**) ATPase assays show that ctPrp22 has an increased ATPase activity compared to ctPrp43, but both are stimulated by the presence of an A_20_-RNA. (**C**) ctPrp22 and ctPrp43 show the same binding mode toward an A_20_-RNA in dependence of an adenosine nucleotide. Both bind the ssRNA best in presence of AMPPNP and show less binding in absence of any adenosine nucleotide. The affinity toward ssRNA is drastically decreased when ADP is present. ATPase activity as well as RNA-binding experiments were determined in triplicates and error bars for each measured data point are depicted. The error of fit is indicated in the tables as +/-.

As in the used construct the N-terminal region, which contains the S1 motif in Prp22, has been completely omitted, only five of the six DEAH-box specific domains are present (50). Two RecA-like domains, referred to as RecA1 and RecA2, build up the helicase core (Figure 1A). These domains harbor the eight conserved sequence motifs known to play a crucial role in ATP and RNA binding as well as in coupling ATP hydrolysis to translocation/RNA unwinding. Apart from these conserved sequence motifs, recent studies on Prp43 and the closely related DExH-box helicase maleless (MLE) have identified additional structural elements, as the hook-loop and hook-turn, which are crucial for the function of these helicases (14,51). The RecA2 domain contains an anti-parallel β-hairpin present in all DExH-box helicases, which protrudes out of the RecA2 domain thereby contacting the C-terminal domains. These domains are divided in three distinct domains, namely the winged-helix (WH), the helix-bundle (HB) and the oligonucleotide-binding (OB) domain. These three domains are arranged on top of the helicase core thereby forming an RNA-binding tunnel between these domains. Upon binding of ATP, this tunnel is exposed and the WH domain serves as a hinge region for the opening of the tunnel (14). Interestingly, the C-terminal 47 amino acids were not traceable in the electron density map and thus are missing in the model of ctPrp22, suggesting a high flexibility of this C-terminal tail. In the most recently published cryo-EM structure of the postcatalytic P complex, the major part of this region could be built, probably due to numerous interactions with other spliceosomal factors and its integration in more inner parts of the complex (25). The global architecture of Prp22 strongly resembles the one already known from Prp2 (PDB ID: 6fa5) and Prp43 (PDB ID: 5lta) (14,37).

The N-terminally truncated Prp22 construct is a competent ATPase and similar to other DEAH-box ATPases it is stimulated by the presence of RNA (Figure 1B) (29,52,53). The degree of stimulation by a single-stranded A_20_-RNA is strongly dependent on the excess given to the ATPase reaction, exhibiting up to a 3.5-fold stimulation of *k*_cat_ and a linear increase of *k*_cat_ and *K*_m_ using up to 40-fold molar excess of ssRNA over protein (Supplementary Figure S1b and c). In contrast to previous findings, reporting that the stimulation by RNA only influences the *k*_cat_, our data shows an increase of both, *k*_cat_ and *K*_m_ (Figure 1B) (21). This discrepancy might be due to the lack of the N-terminal region of the used construct or Prp22 from *C. thermophilum* functions in a different manner than yeast Prp22 in this regard. Prp22 binds ssRNA in absence as well as in presence of adenosine nucleotide (Figure 1C). However, in the presence of the non-hydrolysable ATP-analog AMPPNP, the binding is slightly more efficient than with no adenosine nucleotide present. The binding of ssRNA is significantly decreased in the presence of ADP. Recent structural studies on ctPrp43 indicate that the ssRNA is loaded via an ATP-mediated opening of the RNA-binding tunnel and the increased affinity in presence of AMPPNP is in agreement with these findings (14). Nevertheless, DEAH-box ATPases seem to be able to bind ssRNA also in the absence of ATP, suggesting the opening of the tunnel is not strictly dependent on the binding of ATP but is less efficient without (Figure 1C).

Prp43 is by far the most biochemically and structurally studied spliceosomal DEAH-box ATPase and to ensure mechanistic conclusions derived from structural comparisons in upcoming chapters are legitimate, a comparably truncated version of ctPrp43 (61–764) was subjected to the same basic biochemical characterization. Although *k*_cat_, *K*_m_ and *K*_d_ values deviate from the ones measured for Prp22, both ATPases are stimulated by the presence of RNA and the binding of ssRNA in dependence of the adenosine nucleotide exhibits the same tendencies (Figure 1B and C). Discrepancies in the magnitude of the measured values might arise from the fact that Prp43 depends on the interaction with so called G-patch proteins (Pfa1, Ntr1, Gno1 and Cmg1), which generally have a stimulating effect on Prp43 (22,54,55). Since Prp22 is not known to require a regulatory G-patch protein and has already elevated activity, all biochemical studies as well as structural comparisons were conducted without the consideration of G-patch proteins. Altogether, these findings suggest that these two ATPases share a common mechanism of interacting with ssRNAs, which can be likely applied to the rest of the DEAH-box members, and justifies following structural comparisons of catalytic states of these two ATPases.

### RNA binding of Prp22

**Figure 2.**
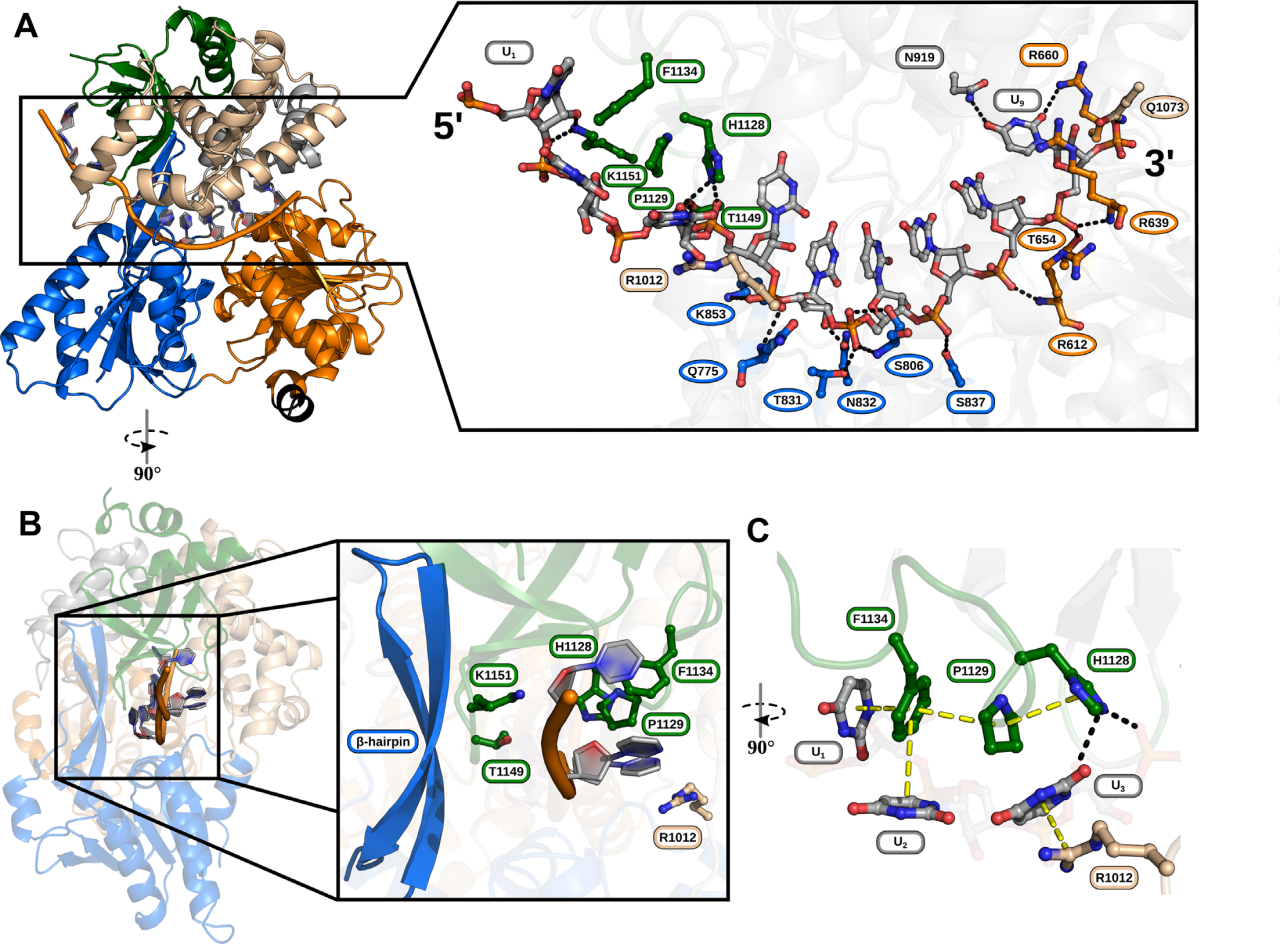
RNA interactions of ctPrp22. (**A**) The ssRNA binds in the RNA-binding tunnel between helicase core and C-terminal domains. The RNA and amino acids either hydrogen-bonding or stacking with nucleotides of the RNA are depicted as ball and stick models and colored according to the domain coloring of Figure 1. The numbering of residues exhibiting the conserved RNA-backbone interaction pattern with the 3′ region as seen as well in the ctPrp43+ADP-BeF_3_^−^+RNA structure (PDB ID: 5lta) is highlighted as ovals, whereas residues displaying a unique interaction have a rectangular shape. (**B**) Overview of 5′ interactions leading to the unique conformation of this region. Bases of U_1_-U_3_ point in the opposite direction of the β-hairpin. Major contributors to this conformation are residues H1128, P1129 and F1134 of the OB-fold domain forming a stacking triad and R1012 from the HB domain (**C**).

For the crystallization of the complex structure of ctPrp22 with a single-stranded RNA, a U_12_-RNA was used, of which nine nucleotides were traceable in the electron density map (Figure 2A). As already reported for other members of the DExH-box family, the ssRNA binds in an RNA-binding tunnel which is formed between the helicase core and the C-terminal domains (14,51,56,57). In contrast to the closely related DExH-box helicases, DHX36 and MLE, which exhibit a preference toward G-rich and U-rich sequences, respectively, genuine DEAH-box ATPases interact with RNA in a sequence-unspecific manner, as has been shown for Prp43 (14). In the Prp22-RNA complex structure, the ssRNA is also predominantly bound via polar interactions with the sugar-phosphate backbone (Figure 2A). The only exceptions are U_9_, as the base forms hydrogen-bond interactions with the side chains of N919 and R660, and the bases of U_1_-U_3_ that interact via π–π-stacking with F1134 and cation–π stacking with R1012 (Figure 2C). The latter interactions are feasible with any base and thus do not determine sequence-specificity.

Amino acids interacting with the sugar-phosphate backbone of RNA-nucleotides U_5_-U_9_ are highly conserved as they are part of the conserved eight sequence motifs of DEAH-box proteins or the recently proposed structural elements hook-loop and hook-turn (Supplementary Figure S2a). Residues R612 (motif Ia), R639 (hook-turn, HT), T654 (motif Ib), T831 (motif V), S837 (motif V) and Q1073 (HB domain) are strictly conserved among DExH-box helicases Prp22, Prp43, MLE and DHX36. K853 (β-hairpin) is replaced by an arginine in MLE still allowing the same type of interaction, S806 (hook-loop, HL) is substituted by a glycine in Prp43 that still interacts with the RNA via the mainchain and N832 is exchanged to an isoleucine in DHX36 from *Drosophila melanogaster* (14,51,56,57) These conserved interactions lead in all structures to a stacked conformation of the RNA in the 3′ region, with the bases pointing toward the C-terminal domains (Figures 2A; 4A and B).

In contrast to the highly conserved backbone interactions and the stacked RNA conformation at the 3′ end, the 5′ end of ssRNAs in complex with DExH-box ATPases exhibits more variability in terms of number of interactions and conformation (Supplementary Figure S2c). While the 5′ end of the RNA only loosely associates with ctPrp43 leading to alternative conformations, the same region of the RNA is highly stabilized in ctPrp22 mainly by stacking interactions with residues of the HB and OB-fold domains (Figure 2B and C) (14). The thorough analysis of the Prp22–RNA complex structure reveals a novel RNA-binding motif present in the OB-fold domain, which involves H1128, P1129 and F1134 as a stacking triad. H1128 directly interacts via hydrogen-bonding with the phosphate of U_4_ and the ribose of U_3_, but also undergoes stacking with P1129, which itself stacks with F1134 (Figure 2C). This last residue of the stacking triad π–π-stacks in a parallel-displaced manner with the U_1_ base as well as in an edge-to-face fashion with the U_2_ base. Additionally, a further cation–π interaction between the U_3_ base and R1012 of the HB domain and hydrogen-bonds between the sugar-phosphate backbone with T1149 and K1151 of the OB-fold contribute to the stabilization of the 5′ end. All these interactions lock the 5′ end of the RNA into a conformation where the bases point into the opposite direction of the β-hairpin of the RecA2 domain (Figure 2B). This is particularly interesting as the β-hairpin has been suggested to be involved in double-strand separation based on the structural comparison with the Ski2-like helicase Hel308 (12,23,31). By orienting the bases of the most 5′ RNA nucleotides away from the β-hairpin, Prp22 likely positions a potential double-strand on the opposite side of this structural feature. This finding is not in agreement with the proposed role of the β-hairpin as it does not seem to act as a strand separator in Prp22.

Spliceosomal cryo-EM structures containing DEAH-box ATPases show that the C-terminal domain of these proteins are the major anchor for the interaction with the spliceosomal complex (25,28,58–61). Especially the OB-fold domain is majorly involved in contacts with other spliceosomal factors and is always facing toward the core of the complexes. This orientation of the ATPases relative to the rest of the complex makes sense as the entrance of the RNA-binding tunnel is already properly positioned for the incorporation of the ssRNA. Here, the stacking triad could help to bridge the distance from the spliceosomal complex to the helicase core of the DEAH-box ATPase and efficiently guide the ssRNA into the binding tunnel. Additionally, during the process of winching, translocation of the DEAH-box proteins is thought to apply pulling forces on the ssRNA and in this case the stacking triad might act as a kind of safety-lock that prevents backlashing of the RNA.

Intriguingly, the histidine and the proline of the stacking triad are strictly conserved among all genuine DEAH-box ATPases, while the phenylalanine is less conserved (Supplementary Figure S2b). In Prp22 from *S. cerevisiae*, it is replaced by a tyrosine and in Prp2 and Prp16 also a histidine or arginine can be found at this position, all of which could still support stacking. Prp43 is the only spliceosomal DEAH-box ATPase, which lacks a residue with comparable property at this position, explaining a discrepant RNA binding mechanism that results in a more flexible binding of the 5′ region (14). The lack of this interaction pattern in Prp43 is likely the reason for the observed decreased binding affinity toward ssRNA (Figure 1C). G-patch proteins interacting with Prp43 have been shown to contact the OB-fold domain and thereby stimulate the basal as well as the RNA-dependent ATPase activity of Prp43 (31,54). During this interaction, the G-patch proteins could potentially provide a residue that completes the stacking triad for the missing amino acid, which could lead to a stimulation of the RNA binding and RNA-dependent ATPase activity.

The differences in the binding of the 5′ end of the ssRNA might play a role in the regulation of the translocase/helicase activity of the different members of this family, whereas the vastly conserved interaction pattern of the 3′ end is required to maintain translocation along a single-stranded RNA as a general mechanism.

### Intrinsic mobility of RecA2 domain enables open conformation of helicase core

**Figure 3.**
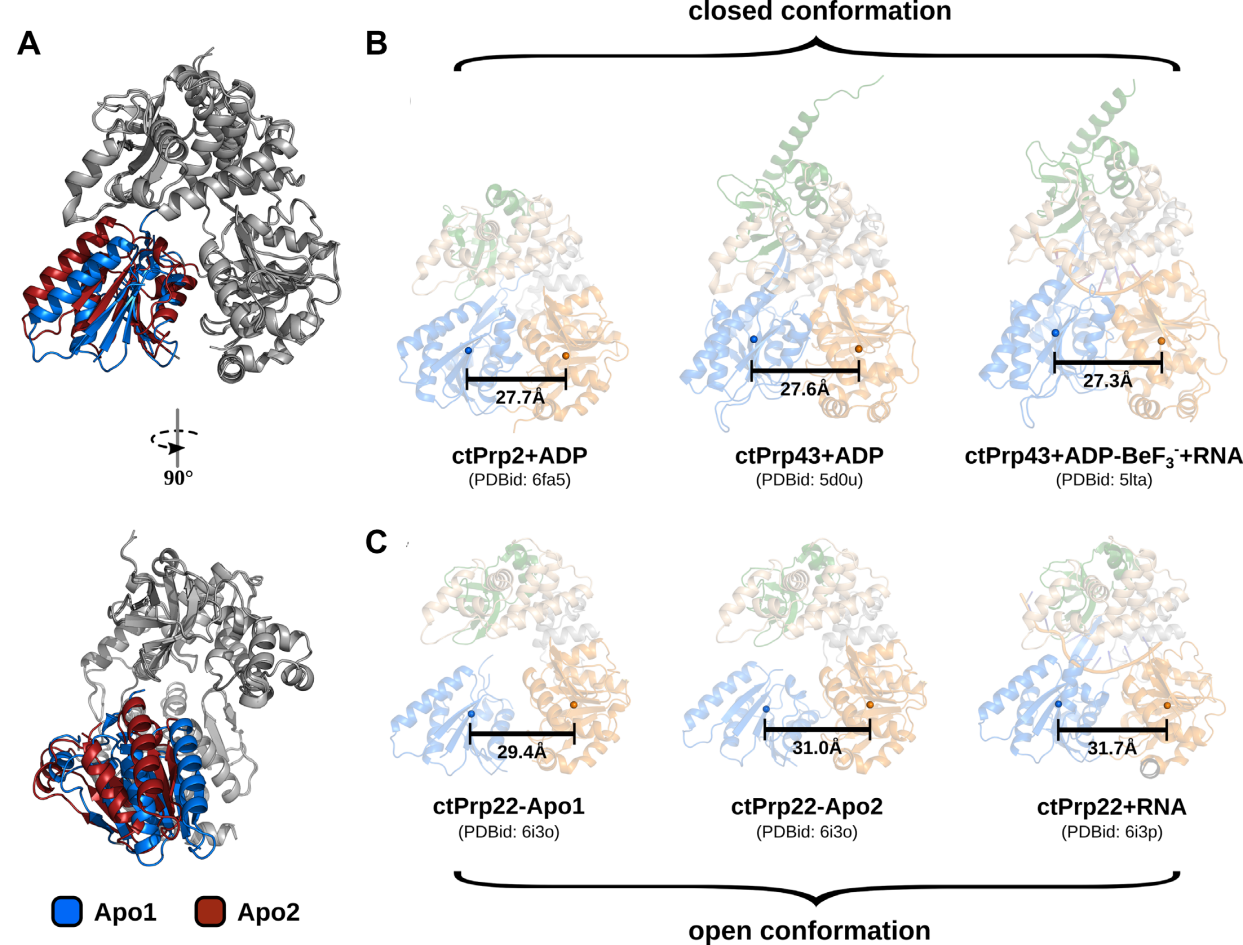
Intrinsic mobility of RecA2 domain and comparison of open and closed conformations of the helicase core. (**A**) Both ctPrp22 molecules found in the asymmetric unit of the Apo structure are depicted as cartoon models. The RecA2 domains are colored in blue (Apo1) and red (Apo2), the RecA1 domains and C-terminal domains are depicted in gray. While the latter domains superpose virtually identically, the RecA2 domains show distinct positions. (**B & C**) All structures are depicted as semi-transparent cartoon models and colored according to Figure 1. Center of mass of the RecA-like domains are displayed as spheres and accordingly colored. In order to calculate the centers of mass for the same sets of atoms, the RecA1 and RecA2 domains of the ctPrp22-RNA complex were superimposed with the corresponding domains of the individual catalytic states and centers of mass for these superposed domains were determined. Adenosine nucleotide-bound DEAH-box ATPase structures adopt a closed helicase core conformation (**B**), whereas ADP/ATP-free structures are found in an open conformation (**C**).

An additional crystal structure of Prp22 in absence of RNA and adenosine nucleotide could be solved (Table 1). The two Prp22 molecules found in the asymmetric unit exhibit virtually identical organization of the RecA1 and C-terminal domains, but strongly differ in the position of the RecA2 domain with respect to rest of the protein (Figure 3A). Although significantly elevated *B*-factors of the RecA2 domain did not allow to build a complete model of this domain (133 of 173 RecA2 residues for Apo1 and 141 of 173 RecA2 residues for Apo2), enough portions of the domain were traceable in the electron density to unambiguously place it with respect to the RecA1 and C-terminal domains (Supplementary Figures S3a and S4a). The two different positions of the RecA2 domain together with the increased *B*-factor values suggest that in absence of any interaction partner this domain has a high degree of freedom to move as a rigid body. In contrast to previously solved adenosine nucleotide-bound structures of genuine DEAH-box ATPases, the RecA2 domains of both Apo structures display an increased distance toward the RecA1 domain (Figure 3B). While the RecA1-RecA2 center of mass distance ranges between 27.3–27.7 Å when ADP/ATP is bound, this distance is increased to up to 31 Å in the unliganded state (14,32,37). The intrinsic mobility of the RecA2 domain allows the helicase core to adopt more open conformations than were previously known from adenosine nucleotide-bound structures.

The presence of ADP/ATP seems to stabilize closed conformations of the helicase core regardless of the availability of RNA, while in the absence of adenosine nucleotides the bound RNA suppresses the mobility of the RecA2 domain and stabilizes the helicase core in an open conformation with a RecA1-RecA2 center of mass distance of 31.7 Å (Figure 3B). *B*-factor values of the RecA2 domain are not elevated anymore in the Prp22–RNA complex structure (Supplementary Figure S3b). As a consequence of the open conformation, the adenosine nucleotide-binding site, which is formed by both RecA-like domains does not exhibit a conformation compatible with high-affinity ATP binding.

Prp22 has been found to be in the open conformation in cryo-EM structures of the C* and post-catalytic P spliceosome complexes and the Prp22–RNA complex crystal structure fits well into the cryo-EM maps of the P complexes published by Liu *et al.* and Wilkinson *et al.* (Supplementary Figure S5a) (25,27,58,62,63). Spliceosomal DEAH-box ATPases Prp16 and Prp43 acting in the C and intron-lariat spliceosome complexes, respectively, were also modeled with an open conformation of the helicase core (60,61,64). Prp2 represents the only exception, as it has been modeled in the ADP-bound state and thereby closed conformation in two B^act^ complex cryo-EM structures (28,59). A fit of the Prp22–RNA complex into the Prp2 cryo-EM map within the B^act^ complex published by Yan *et al.* shows that an open conformation of Prp2 would be in better agreement with the data (Supplementary Figure S5b). A caveat of spliceosomal cryo-EM structures up to date is the significant drop in resolution toward peripheral regions of the complex (65). Since all DEAH-box ATPases are located at the outer rim of the spliceosomes, the resolution for these proteins is low and usually no active-site nucleotide can be identified to accurately assign the catalytic state of the ATPase. However, the information of the adenosine nucleotide-specific domain arrangements known from crystal structures can be used to improve the interpretation of DEAH-box ATPases in spliceosomal cryo-EM structures. The presented adenosine nucleotide-free/open ctPrp22 structures complement the previously known adenosine nucleotide-bound/closed DEAH-box ATPase structures and complete the structural repertory of adenosine nucleotide-specific catalytic states.

### DEAH-box ATPases translocate at a step-size of one RNA nucleotide per hydrolyzed ATP

**Figure 4.**
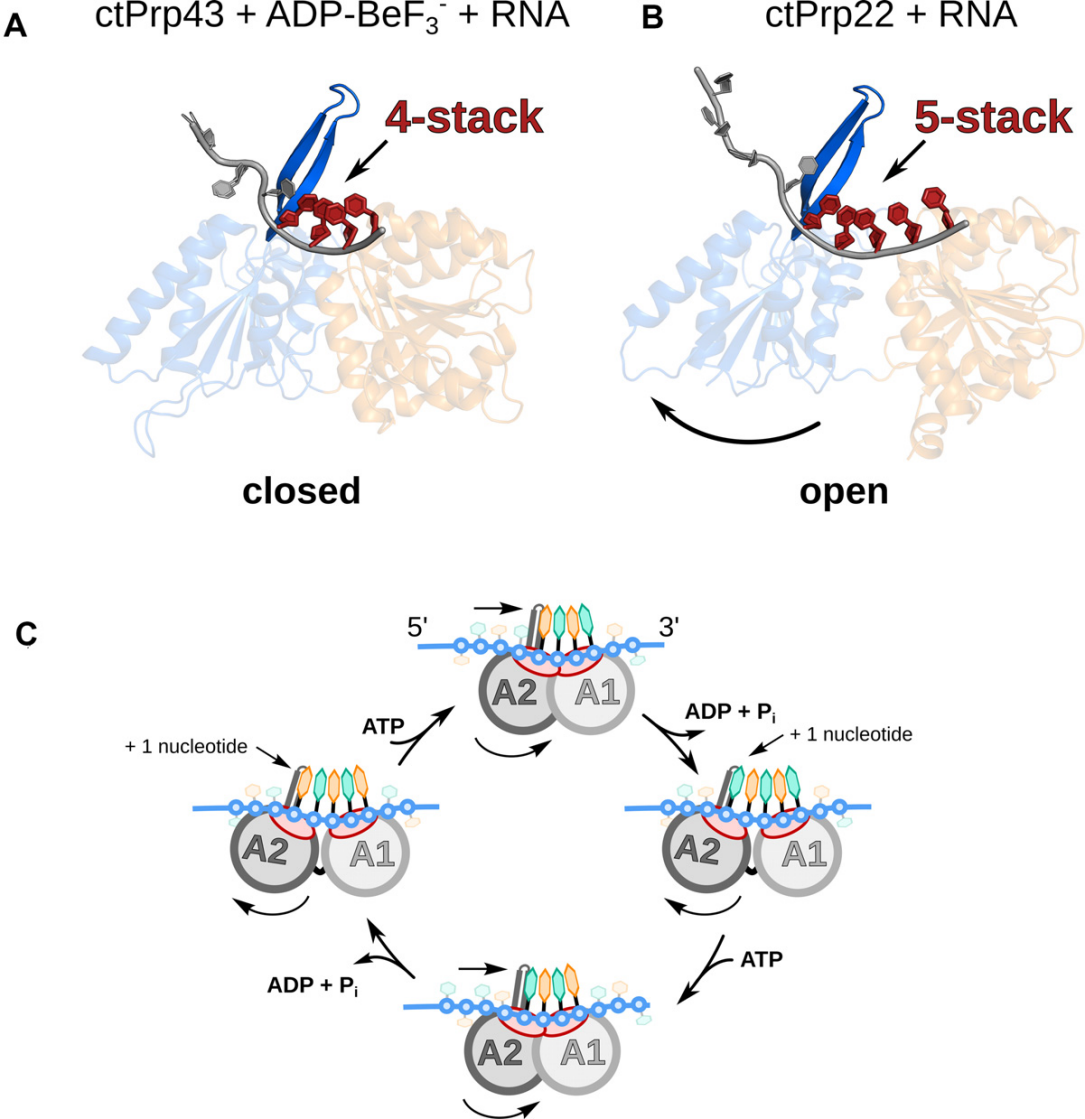
DEAH-box ATPases translocate at a step-size of one RNA nucleotide per hydrolyzed ATP. The helicase cores of ctPrp43 and ctPrp22 with bound ssRNAs are depicted as cartoon models with a domain coloring according to Figure 1. (**A**) ATP-bound ctPrp43 exhibits a closed conformation of the helicase core with a stack of four RNA nucleotides bound to the RecA-like domains. (**B**) In the absence of an adenosine nucleotide the helicase core of ctPrp22 adopts an open conformation, which allows the accommodation of an additional RNA nucleotide in the binding tunnel leading to a bound five-nucleotide stack. In both conformations, the stack is interrupted by the β-hairpin of the RecA2 domain. (**C**) A continuous cycling of these states enables DEAH-box ATPases to translocate in 3′ to 5′ direction along a single-stranded RNA. Upon helicase core opening, an additional RNA nucleotide can be incorporated between the β-hairpin and the first RNA nucleotide of the four-nucleotide stack. The helicase core closure induced by the binding of ATP pushes the RNA through the binding tunnel.

Crystallographic studies on DExH-box ATPases over the last decade have revealed that this protein family is able to adopt a variety of conformations and that these structural rearrangements vary as a function of the ligand bound (ATP-bound state, ADP-bound state, adenosine nucleotide-free but RNA-bound state and Apo) (13,14,23,31,32,37,51,56,57). Ligand-specific arrangements of the RecA-like domains are structurally conserved regardless of species and member of the DExH-box family. ADP- and ATP-bound structures of Prp43 as well as adenosine nucleotide-free but RNA-bound structures of DHX36 exhibit virtually identical helicase core conformations in different species (Supplementary Figure S6a–c). These conformations are also conserved among different subfamily members since the helicase cores of Prp2, MLE and Prp22 align well with the corresponding structures of Prp43 and DHX36 (Supplementary Figure S6a–c). Combining these structurally conserved catalytic steps, we propose the following model for the single-stranded RNA translocation of DEAH-box ATPases:

In the closed conformation of the helicase core, the adenosine nucleotides are always sandwiched between both RecA-like domains, thereby providing an additional interaction network tightly connecting the two domains (13,14,23,31,32,37). When ATP is bound, this closed conformation allows the conserved sequence motifs Ia, Ib, IV and V as well as the hook-loop, the hook-turn and the β-hairpin to interact with the 3′ region of the ssRNA (Supplementary Figure S7a). The transition from ATP to ADP after hydrolysis rearranges the ATP-mediated interaction network bridging RecA1 and RecA2, which induces a reorientation of the domains. The RecA2 domain is rotated by 19° with a rotation axis close to the center of mass as it is only minimally displaced by 1.8 Å (Supplementary Figure S8b and Supplementary Movie SM1). Up to date, only ADP-bound structures in absence of RNA could be solved and no accurate model of an ADP- and RNA-bound DEAH-box ATPase is available. However, by modeling the ssRNA of the ATP-bound structure into the ADP-bound structure via a superposition of the RecA1 domains, the conserved RecA2 residues interacting with the RNA are displaced by 2.6–4.6 Å (Supplementary Figure S7c and d). This displacement results in a loss of contact of the RecA2 domain with the RNA, which is in good agreement with the observed reduced affinity toward RNA in the presence of ADP (Figure 1C). After the release of the ADP, all nucleotide-mediated interactions between both RecA-like domains are lost. Together with the loss of contact of the RecA2 domain with the RNA backbone, this probably enables a complete detachment of the RecA2 domain and allows independent movement as implied by the Prp22 Apo structure (Figure 3A). This positional freedom is suppressed by the interactions of the conserved sequence motifs of the RecA2 domain with the RNA backbone, which shifts this domain by one RNA nucleotide toward the 5′ end (Supplementary Figure S7b). While the DEAH-box ATPase encloses a stack of four RNA nucleotides in the closed and ATP-bound state, this open and adenosine nucleotide-free conformation accommodates a five-nucleotide stack in the RNA-binding tunnel (Figure 4A and B). During the transition from the closed to the open conformation, the RecA2 β-hairpin is shifted enough to allow the incorporation of the fifth RNA nucleotide between the preformed four-nucleotide stack and this structural motif (Figure 4C and Supplementary Movie SM2). In both conformations, the β-hairpin serves as a physical barrier for the stack and redirects the RNA allowing the 5′ end to interact primarily with the C-terminal domains (Figure 2). Upon ATP-binding to the RNA complex, the RecA2 domain is shifted by 5.8 Å, bringing both RecA-like domains back into close proximity (Supplementary Figure S8b). The movement of the RecA2 domain toward the RecA1 domain pushes the ssRNA in 3′ direction through the binding tunnel, spanning a distance equivalent to one RNA nucleotide (Figure 4C). Due to the higher amount of interactions of the RecA2 domain with the RNA backbone (RecA2: 6 *versus* RecA1: 3) and the mechanical aid of the β-hairpin, the RNA stays stably attached to the RecA2 domain and does not backlash. Instead, the few RecA1 interactions need to be broken and subsequently replaced by the succeeding sugar-phosphates pushed into that position. A cycling of the described events enables DEAH-box ATPases to translocate along an ssRNA in 3′-5′ direction at a step-size of one RNA nucleotide per hydrolyzed ATP (Figure 4C; Supplementary Movies SM1 and SM3).

### Helicase core rearrangements are driven by a serine in motif V that senses catalytic state

**Figure 5.**
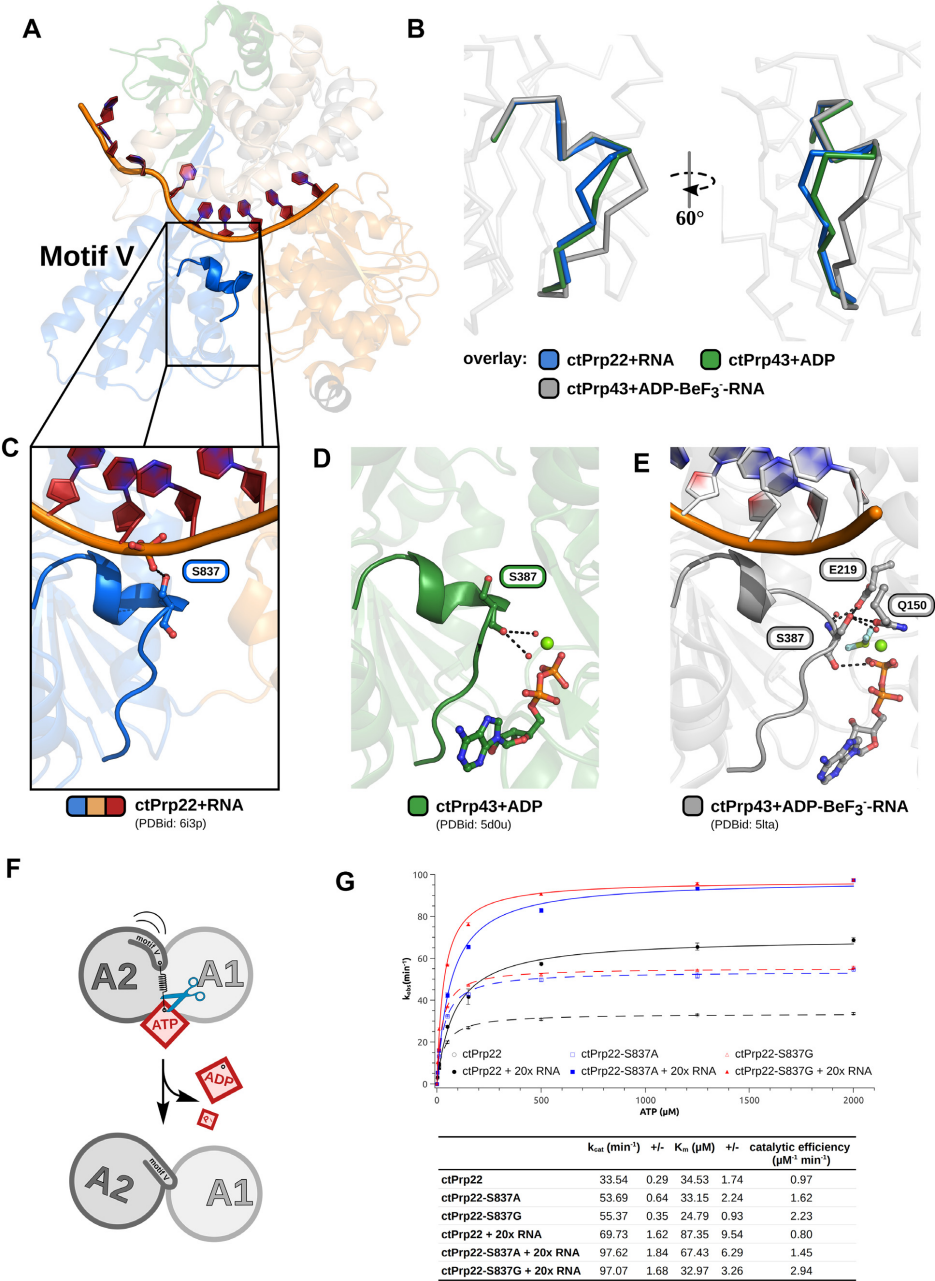
A serine in motif V senses the catalytic state. (**A**) Motif V is located in the RecA2 domain in close proximity to the RNA-binding tunnel and the active site. (**B**) Motif V exhibits a helical conformation in the absence of adenosine nucleotides, as well as when ADP is bound. In the presence of ATP, the helix is disrupted and the motif adopts an alternative conformation. A serine in this motif senses the current catalytic state via different polar interactions with the bound ligands (**C–E**). In the adenosine nucleotide-free but RNA-bound state, the serine side chain hydrogen bonds with an ssRNA phosphate (C), and in the presence of ADP, the mainchain interacts with two water molecules coordinated by the active site magnesium (D). (E) When ATP is bound, the motif V helix is distorted by the interactions of the serine with numerous active site components. The mainchain interacts with the catalytic water molecule and a magnesium-coordinated water molecule. The side chain undergoes polar interactions with an active site glutamate and glutamine as well as with another water molecule coordinated by the magnesium. (**F**) The ATP-dependent interactions of the serine force motif V into an unfavored conformation. Upon ATP hydrolysis the motif is able to relax back into its helical conformation, which is translated into a repositioning of the RecA2 domain. (**G**) ATPase activity measurements of ctPrp22-S837A and ctPrp22-S837G mutants compared with wild-type ctPrp22 in absence and in presence of A_20_-ssRNA. The experiments were determined in triplicates and error bars for each measured data point are depicted. The error of fit is indicated in the table as +/-.

In order to ensure the right positioning of the RecA2 domain at any catalytic step needed for translocation, DExH-box members require a mechanism to sense the current ligand-bound state. The conserved sequence motif V has already been suggested to play an important role in the transition between catalytic states, but up to date no detailed molecular mechanism has been proposed for its function (10,11,13). Motif V is a structural element within the RecA2 domain and it is located in the interface between the adenosine nucleotide-binding site and the RNA-binding tunnel (Figure 5A). At this position it is in close proximity to the bound ADP/ATP and ssRNA. In the Apo, ADP-bound and RNA-bound structures this motif forms a short α-helix, which is partially distorted when ATP is bound (Figure 5B–E; Supplementary Figure S9a and Supplementary Movie SM4). A structural analysis revealed that a serine in motif V is involved in polar interactions with one of the ligands in all structurally characterized catalytic states. In the adenosine nucleotide-free but RNA-bound state, the side chain of the serine contacts the U_7_ phosphate and thereby bridges the gap that is formed by the more distant conformation of the RecA-like domains (Figure 5C). In the ADP/ATP-bound structures, the mainchain of this residue interacts via hydrogen-bonds with conserved water molecules constituting the active site (Figure 5D and E). The side chain of the serine additionally interacts with another water molecule and two active site residues when ATP is present (Figure 5E). The interactions in the ATP-bound state force the serine mainchain to adopt an unfavored conformation, enabling the mainchain nitrogen to hydrogen bond with the catalytic water (Supplementary Figure S9d). As a consequence, the C-terminus of the motif V helix is disrupted (Figure 5B). Upon ATP hydrolysis, the interactions at the active site are rearranged and the serine looses its hydrogen bond network allowing the serine mainchain and the entire motif V to relax into the energetically more favorable helical conformation. This contributes to a global repositioning of the RecA2 domain, since the helical motif V conformation would clash with motif Ia of the RecA1 domain in the ATP-bound helicase core arrangement (Figure 5F and Supplementary Figure S9e). This steric hindrance forces the RecA2 domain to interact with RNA nucleotides positioned one residue further to the 5′ end when no adenosine nucleotide is present and thereby achieving the one RNA nucleotide step-size. Such a mechanism shows parallels to the loaded-spring model of G domain proteins and ATP-dependent motor proteins like kinesin and myosin (66–68). Here, the so-called conserved switch regions act as γ-phosphate sensors that are released in a spring-like manner upon NTP hydrolysis, triggering conformational changes of other domains (69).

In order to verify the central role of this serine, we created alanine, glycine and proline mutants of this residue in Prp22 and characterized their ATPase activity (Figure 5G). The lack of the hydroxyl group in Prp22-S837A mainly leads to an increase of *k*_cat_ regardless of the presence of RNA and only to a minor decrease of *K*_m_ when RNA is present. In both cases, the catalytic efficiency (*k*_cat_/*K*_m_) is improved by a factor of ∼1.7. Since mainly the mainchain of the motif V serine interacts via water-mediated hydrogen bonds with the adenosine nucleotides, we tested if a higher flexibility of the mainchain at this position has an effect on catalysis. The substitution by a glycine in Prp22-S837G has no significant effect on *k*_cat_ compared to the alanine mutant, but significantly improves the *K*_m_, especially in the presence of RNA. The catalytic efficiency increases 2.3-fold in the absence of RNA and 3.7-fold in the presence of RNA. The gain in flexibility at this position in motif V appears to improve the binding/sensing of ATP rather than stimulating the turnover rate. In contrast to an increase in flexibility of the mainchain at this position, we tested a decrease in flexibility by mutating the serine to a proline. Prp22-S837P did not show any significant ATPase activity (data not shown). In order to exclude misfolding as a reason for the lack in activity, we subjected Prp22 and Prp22-S837P to circular dichroism spectroscopy measurements and could verify the structural integrity of Prp22-S837P (Supplementary Figure S9f). We could furthermore structurally characterize Prp22-S837A in the adenosine nucleotide-free but RNA-bound state, as well as Prp43-S387A and Prp43-S387G in the ATP-bound state (Table 1). Since Prp22 could not be crystallized in the presence of an ATP-analog, an existing crystallization condition for ADP-BeF_3_^−^-bound Prp43 was used to structurally characterize motif V serine mutants of a DEAH-box ATPase in this state (14). The structures of these three mutants show that motif V is able to adopt the same nucleotide-specific conformations regardless of the side chain property (Supplementary Figure S9b and c).

Summarizing our data show that the serine side chain is dispensable for the function of motif V and instead has a regulatory role. The polar interactions of the hydroxyl group with the RNA phosphate and active site components seem to negatively regulate the turnover rate of DEAH-box ATPases. Instead, the mainchain is sufficient for sensing the adenosine nucleotide state at the active site and this sensing function is sensitive to the flexibility of the mainchain. While the ATP binding is improved at maximum flexibility (glycine), the sensing function is completely abolished at minimum flexibility (proline).

## CONCLUSIONS

In eukaryotes non-coding introns in precursor mRNAs are excised via the spliceosome. One key player during pre-mRNA splicing is the DEAH-box ATPase Prp22, which is critical for the release of mature mRNAs. In this study, we determined the first crystal structures of the spliceosomal DEAH-box ATPase Prp22 in the adenosine nucleotide-free but RNA-bound state, as well as in absence of any ligand. Prp22 shares a conserved architecture of the catalytic unit with other spliceosomal DEAH-box ATPases and interacts with RNA in a comparable manner as Prp43. In the absence of RNA and adenosine nucleotide, the RecA2 domain has an increased ability to move independently as a rigid body and a serine in motif V ensures the proper positioning of this domain in dependence of the bound ligand state. A comparison with the ATP-bound state of Prp43 shows that these ATPases toggle between a closed conformation of the helicase core, which accommodates a stack of four nucleotides in the RNA-binding tunnel, and an open conformation with a bound five-nucleotide stack. A cycling of these events enables these proteins to translocate in 3′-5′ direction along a single-stranded RNA with a step-size of one RNA nucleotide per hydrolyzed ATP (Supplementary Movie SM1 and SM3). The proposed model might also provide an explanation for the RNA-dependent ATPase stimulation as a common feature of DEAH-box ATPases. In the RNA bound but adenosine nucleotide-free structure, the RecA2 domain is fixed by the RNA in a conformation distant enough from the RecA1 domain to provide good accessibility for the binding of ATP, but close enough to directly sense the bound ATP by the motif V serine and induce helicase core closure. In contrast, without bound RNA the RecA2 domain is not optimally prepositioned due to its intrinsic mobility, leading to a prolonged ATP sensing time.

Two different models for the translocation of DEAH-box ATPases have been proposed in the past (13,14,51). The contraction model suggests a continuous transition between a three- and a four-nucleotide stack in the RNA-binding tunnel, caused by primarily smaller rotational movements of the helicase core (13). However, the open helicase core conformation of Prp22 with a stack of five RNA nucleotides strongly argues in favor of the expansion model (13). This model can most likely be extended to other genuine DEAH-box members as they are all highly conserved in sequence and structure. In fact, this translocation mechanism seems to be applicable to the entire DExH-box family, since the recently solved structure of nucleotide-free but nucleic acid-bound DHX36 shows a similar open conformation as the ctPrp22+RNA complex (Supplementary Figures S6c and S8a) (56,57). DExH-box members, DHX36 and MLE, even harbor an almost identical motif V to drive the opening and closing of the helicase core.

Even for more distant relatives of the SF2 helicase family this translocation mechanism seems to be conserved. Using structural and biophysical approaches like smFRET a translocation step-size of as well one RNA nucleotide could be determined for NS3 helicases (70–72). These helicases also translocate along an ssRNA by repeatedly cycling between open and closed conformations of the helicase core upon continuous ATP hydrolysis (Supplementary Figure S10a and b). While the architecture of the helicase core seems to be highly conserved between DExH-box and NS3 members, the C-terminal domains differ significantly (Supplementary Figure S11). Due to the much smaller C-terminal domain, NS3 helicases possess a shorter RNA-binding tunnel that only accommodates the stacked RNA region (Supplementary Figure S10). NS3 helicases thus lack an extensive interaction network with the C-terminal domain as seen for the 5′ end of the ssRNA in complex with Prp22. This independence from a C-terminal domain completely decouples the translocation mechanism primarily driven by the RecA-like domains from any potential regulatory function this additional 5′-interaction might have in DEAH-box ATPases. It also leads to a different trajectory of the RNA through the tunnel accompanied by a divergent position of the RecA2 domain, especially in the ATP-bound state (Supplementary Figure S10c and d). While the adenosine nucleotide-specific helicase core arrangement slightly differs among these two SF2 members, the mechanism to sense the nucleotide state and accordingly position the RecA2 domain is likely conserved. Despite the difference in sequence conservation, a structurally conserved motif V is also present in NS3 helicases (Supplementary Figure S12a–c). The proposed motif V sensing mechanism also applies to NS3 helicases as this motif also switches from a helical toward a distorted conformation in an adenosine nucleotide-dependent manner (Supplementary Figure S12a and b). Interestingly, the sensor serine of DExH-box ATPases is replaced by a glycine in NS3 helicases and more distant relatives like the Ski2-like helicases and even in members of the SF1 family (Supplementary Figure S12c). The lack of a side chain at this residue position in motif V of other SF2 members is in agreement with the findings that it is dispensable for the function. The increased flexibility of a glycine at this position in other SF2 members might be required to ensure the sensing mechanism due to the different helicase core conformations. Apart from the described structural differences, NS3 helicases seem to show also some mechanistic differences. While in NS3 helicases the ATP-bound state is defined as the low-affinity state and the adenosine nucleotide-free state as the high-affinity state towards ssRNA, DEAH-box ATPases exhibit rather the opposite (Figure 1C). Summarizing, while the motif V driven helicase core rearrangements of DExH-box ATPases and NS3 helicases show parallels in agreement with the expansion model for translocation, differences in size and property of the C-terminal domains might have a regulatory impact causing structural and mechanistic variations among these SF2 members. It also remains unclear if the expansion mechanism shared by DExH-box and NS3 members is more universally conserved and could apply as well to other members of the SF2 family known to depend on translocation, like the Ski2-like helicases (12).

## DATA AVAILABILITY

The coordinates and structure factors have been deposited in the PDB, with following accession numbers:

- 6I3O - ctPrp22 Apo
- 6I3P - ctPrp22 + RNA
- 6QIC - ctPrp22-S837A + RNA
- 6QID - ctPrp43-S387A + ADP-BeF_3_^−^
- 6QIE - ctPrp43-S387G + ADP-BeF_3_^−^

## Supplementary Material

Supplementary Data
