# Supplementary material for: Structural basis for RNA translocation by DEAH-box ATPases
Source: Nucleic Acids Res. 2019 Mar 4;47(8):4349–62. doi: 10.1093/nar/gkz150 (PMC6486627; doi:10.1093/nar/gkz150)
Supplement: Supplementary Data [file gkz150_supplemental_files.zip › Supplements+Legends-new-compress.pdf]

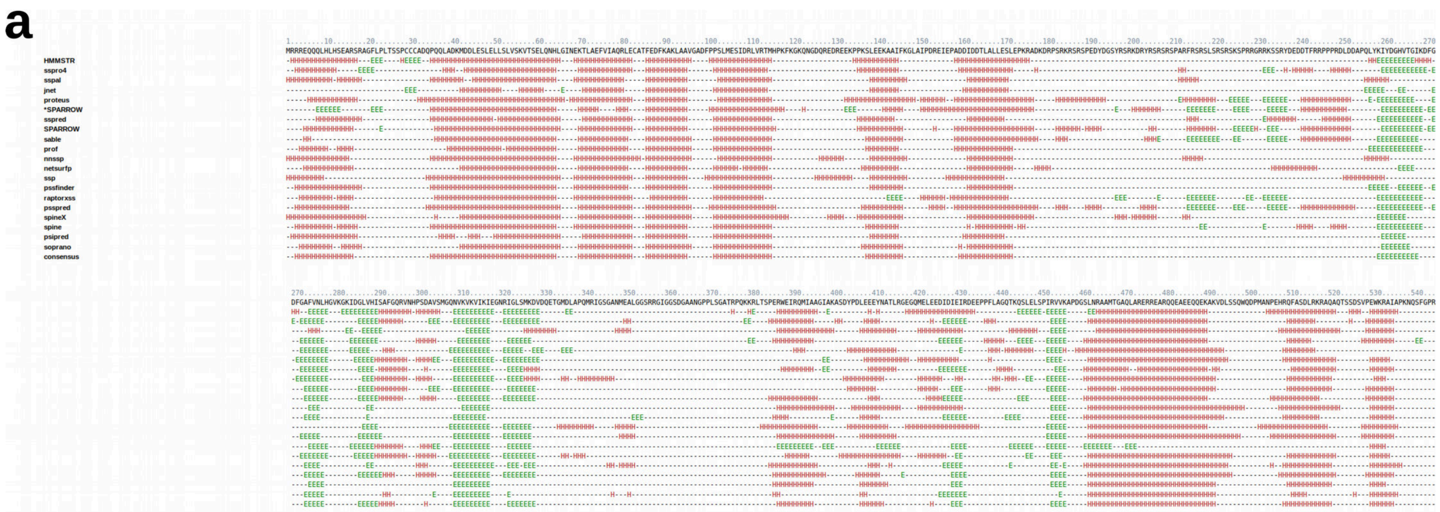

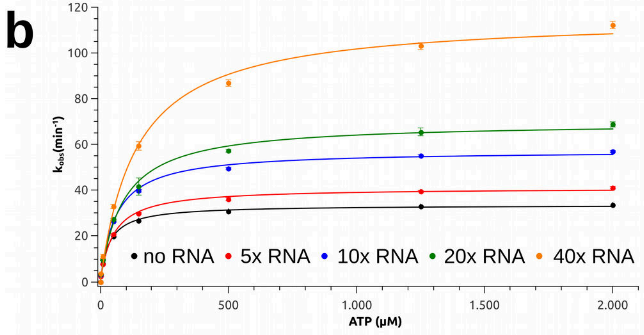

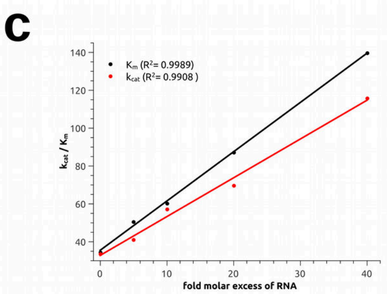

	k_{cat} (min^{-1})	+/-	K_m (µM)	+/-	catalytic efficiency (µM^{-1} min^{-1})
**ctPrp22**	33.54	0.29	34.53	1.74	0.97
**ctPrp22 + 5x RNA**	41.03	0.57	50.67	3.72	0.81
**ctPrp22 + 10x RNA**	57.32	0.85	60.36	4.55	0.95
**ctPrp22 + 20x RNA**	69.73	1.62	87.35	9.54	0.80
**ctPrp22 + 40x RNA**	115.97	2.64	139.65	13.57	0.83

Supplemental figure S1

Secondary structure prediction of the N-terminus of *Chaetomium thermophilum* Prp22 and analysis of RNA-dependent stimulation of ATPase activity of ctPrp22. (a) The GeneSilico metaserver was used to predict the secondary structure of the N-terminal extension of ctPrp22 (Kurowski and Bujnicki 2003). Individual prediction server are stated first, followed by the corresponding prediction to the right. Residues predicted as part of α-helices are highlighted by red “H” and residues predicted as part of β-strands are displayed as green “E”. (b) To examine the RNA-dependent stimulation of ctPrp22, the ATPase activity was measured at different RNA concentrations up to 40-fold molar excess. k_{cat} and K_m are stimulated in a linear fashion by increasing concentrations of RNA. At 40-fold molar excess ctPrp22 exhibits a 3.5-fold stimulation compared to the ATPase activity in absence of RNA.

a

Residue	Atom	Nucl.	Atom	ct/scPrp43	MLE	dm/bsDHX36	motif
R612	N	8	OP1	R153/150	R443	R212/R268	Ia
R639	N	9	OP1	R180/177	R470	R239/297	HT
T654	OG1	9	OP1	T195/192	T486	T255/T313	Ib
R660	NE2	9	O2	R201/198	R492	Q261/Q319	Ib
Q775	N	5	OP2	E316/313	W663	Y432/W500	IV
S806	N	6	OP1	***G349/346***	S692	S464/S528	HL
S806	OG	6	OP2	***G349/346***	S692	S464/S528	HL
T831	OG1	6	OP1	T381/376	T717	T489/T553	V
N832	OD1	5	O2‘	N382/376	N718	***I490***/N554	V
S837	OG	7	OP1	S387/382	S723	S495/S559	V
K853	NZ	5	OP1	K403/398	***R739***	K511/K575	BH
N919	ND2	9	O4	*N469/S464*			
R1012	stack	3	base	R562/557			
Q1073	NE2	10	OP2	Q627/622	Q966	Q736/Q802	
H1128	NE2	3	O4‘	H688/682	H1032	H809/H872	
H1128	NE2	4	OP2	H688/682	H1032	H809/H872	
F1134	stack	1	base	/			
F1134	stack	2	base	/			
T1149	OG1	4	OP1	T708/702			
K1151	NZ	1	O3‘	K710/704			
K1151	NZ	1	O2‘	K710/704			

b

```
ctPrp22   1121 EGTPVYLHPSSALFG.......KQAEWVLY 1143
scPrp22   1047 GGTEVGIHPSSSLYG.......KEYEYVMY 1069
hsPrp22   1129 DQQVVYIHPSSALFN.......RQPEWVVY 1151
ctPrp43    681 DEQLVLIHPSTTV.T.......TPYEWVVY 702
scPrp43    675 DNQDVLIHPSTVL.G.......HDAEWVIY 696
hsPrp43    715 DNQVVQLHPSTVL.D.......HKPEWVLY 736
ctPrp2     870 NNITVYVHPSSVVRGM.....DPPPKVIIY 894
scPrp2     803 GGLNVSVHPTSILFVNHKEKAQRPSKYVLY 832
hsPrp2     964 QQQTVFIHPNSSLFE.......QQPRWLLY 986
ctPrp16    871 TSVTVQLHPTSALYGL.....GFLPDYVVY 895
scPrp16    929 TGVSVQLHPTSALHGL.....GDLPPYVVY 953
hsPrp16   1095 TGMPCHLHPTSSLFGM.....GYTPDYIVY 1117
```

c

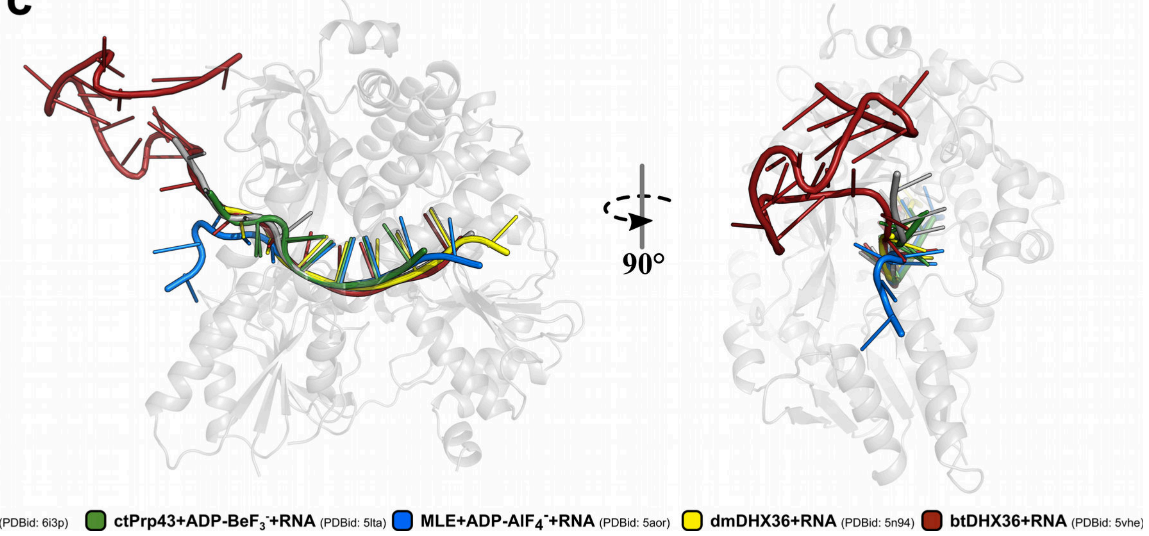

Supplemental figure S2

RNA interactions of DExH-box ATPases. (a) Overview of all residues involved in RNA interactions in the ctPrp22+RNA complex, colored according to figure 1. Conserved residues of other structurally characterized DExH-box ATPase-RNA complexes are listed and exceptions are highlighted in bold/italic. (b) Sequence alignment of the stacking triad of all spliceosomal DEAH-box ATPases from *Chaetomium thermophilum* (ct), *Saccharomyces cerevisiae* (sc) and *Homo sapiens* (hs). The histidine and proline are strictly conserved, but the phenylalanine of ctPrp22 is replaced by other residues potentially able to perform a comparable stacking in scPrp22, ctPrp2 and Prp16. In Prp43 no residue with such a property can be found at this position. (c) Conformational comparison of different RNA-containing DExH-box ATPase complexes. All complexes were superimposed *via* the RecA2 domain and only ctPrp22 is displayed as a semi-transparent cartoon model. ssRNAs of the different complexes show a highly similar conformation of the stacked 3' region, but they strongly differ in the 5' region due to non-conserved interactions.

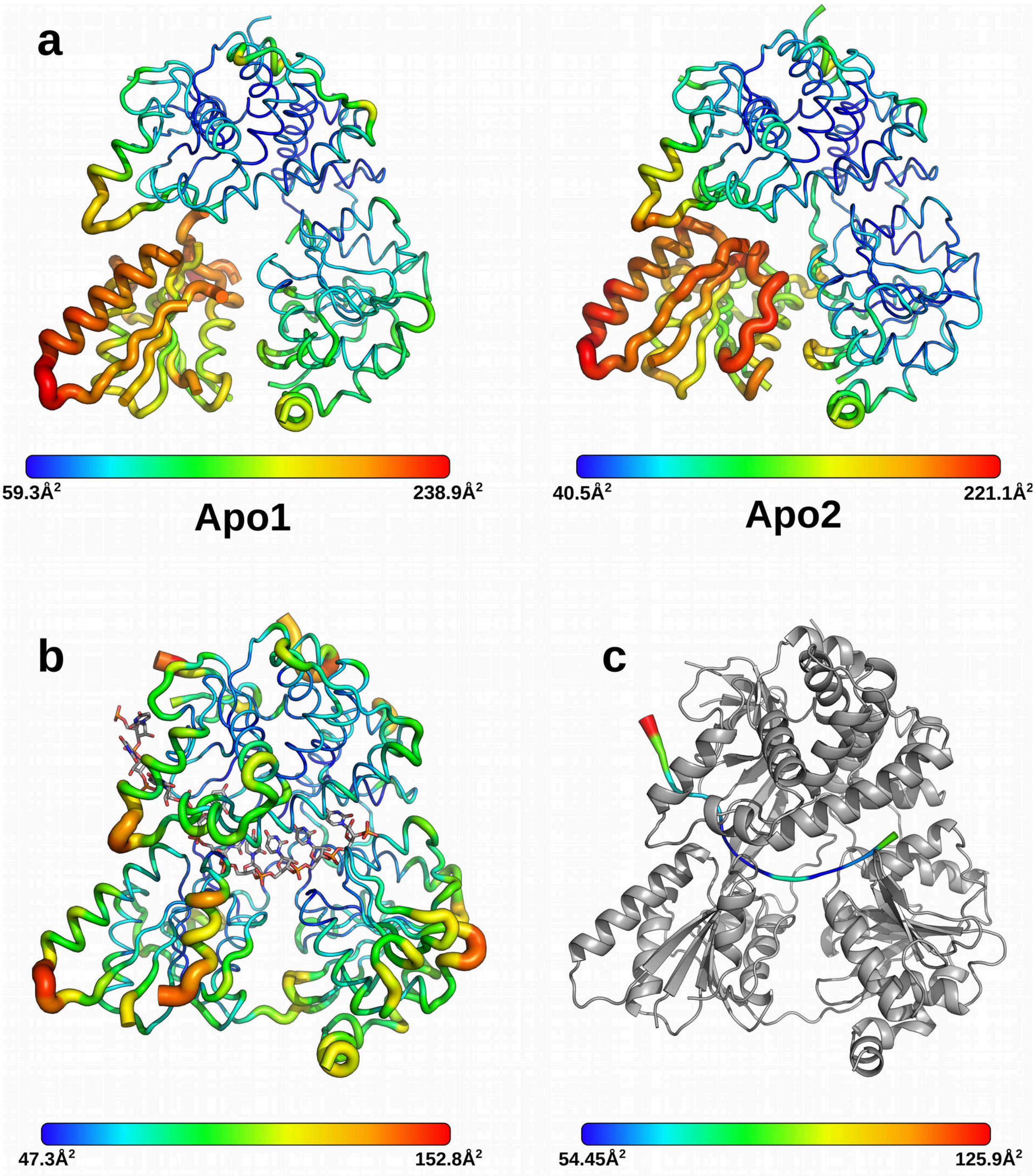

d

	ctPrp22 Apo	ctPrp22 + RNA
**A – B**	2.63* (570**)	0.51 (621)
**A – C**	/	0.41 (625)
**A – D**	/	0.39 (626)
**B – C**	/	0.42 (623)
**B – D**	/	0.50 (623)
**C – D**	/	0.42 (629)
**RecA1 + CTD**	0.46 (442)	/
**RecA2**	0.91 (127)	/

* r.m.s.d. in Å

** C_α-atoms used for superposition

Supplemental figure S3

***B*-factor distribution of the ctPrp22 structures and r.m.s.d. values of all chains of ctPrp22 Apo and ctPrp22+RNA.** (a) The overview representation of the *B*-factors highlights the mobility of the RecA2 domain. (b) All domains of ctPrp22 show an equal distribution of *B*-factors when ssRNA is bound. (c) The ssRNA exhibits only elevated *B*-factor values for the most 5' phosphate but consistently low values for the RNA-nucleotides integrated in the binding tunnel. (d) List of r.m.s.d. values of pairwise alignment of all chains in ctPrp22 Apo and ctPrp22+RNA crystal structures.

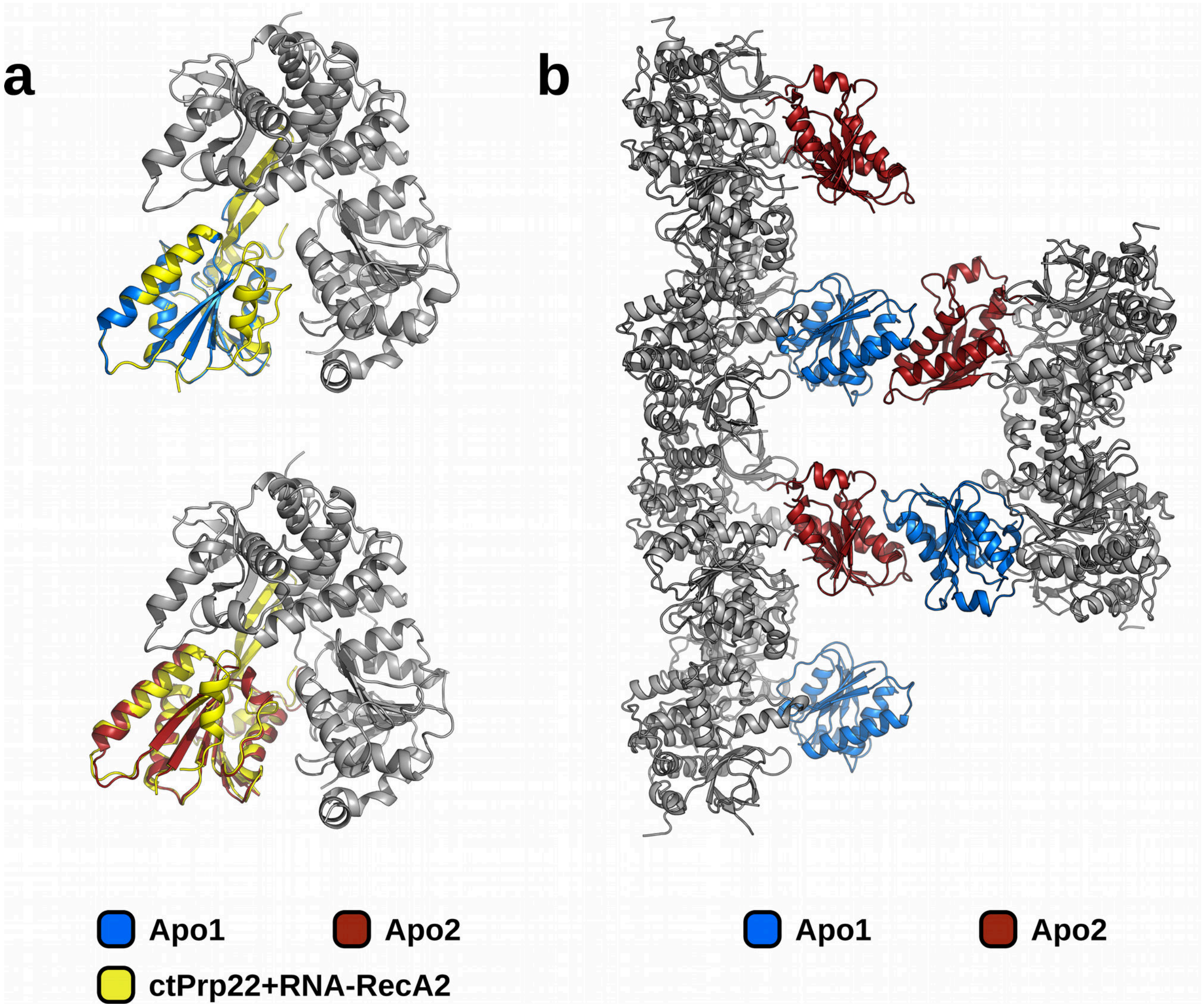

Supplemental figure S4

Mobility of RecA2 domain. ctPrp22 models are depicted as in figure 3a. Due to the property of the RecA2 domain to move flexibly as a rigid body, *B*-factors were elevated to a degree that did not allow to build a complete model of this domain. (a) shows a superposition of the complete RecA2 domain of the ctPrp22+RNA complex (yellow) on the RecA2 domains of both Apo molecules. (b) The crystal packing of the ctPrp22-Apo structure shows that the RecA2 domain undergoes the majority of its crystal contacts with the RecA2 domain of the symmetry related molecule. This does not lead to an effective stabilization of the domain further explaining the observed elevated *B*-factor values.

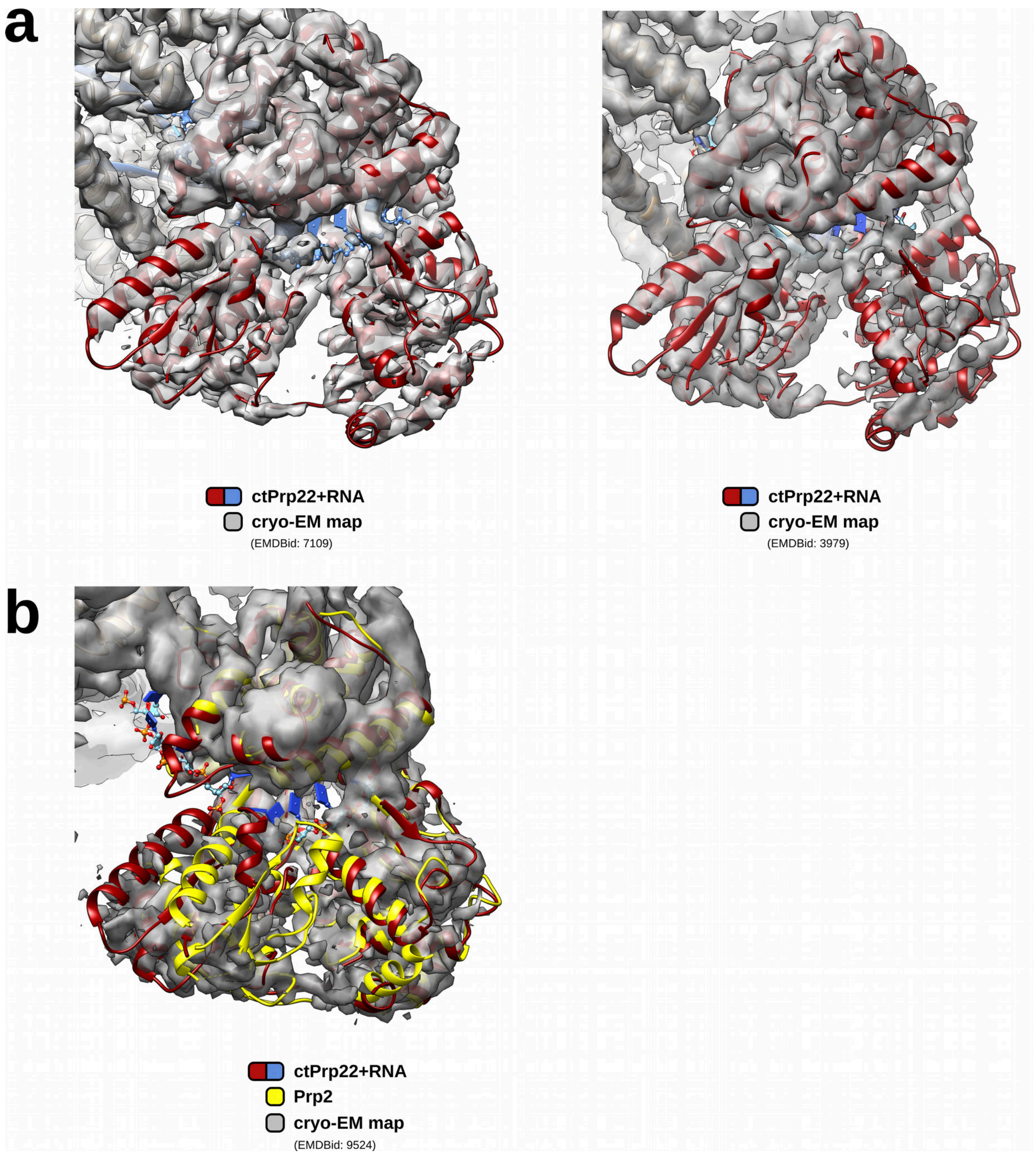

<u>**Supplemental figure S5**</u>

Fit of ctPrp22+RNA structure into cryo-EM maps of the yeast spliceosomal postcatalytic P and B^{act} complexes. The open conformation of the RNA-bound (blue) ctPrp22 (red) complex fits well the data of the yeast spliceosomal postcatalytic P complexes (a) (Liu et al., 2017; Wilkinson et al., 2017) and B^{act} (b) (Wan et al., 2017) complex helping to assign a specific catalytic state of the ATPase for these cryo-EM structures.

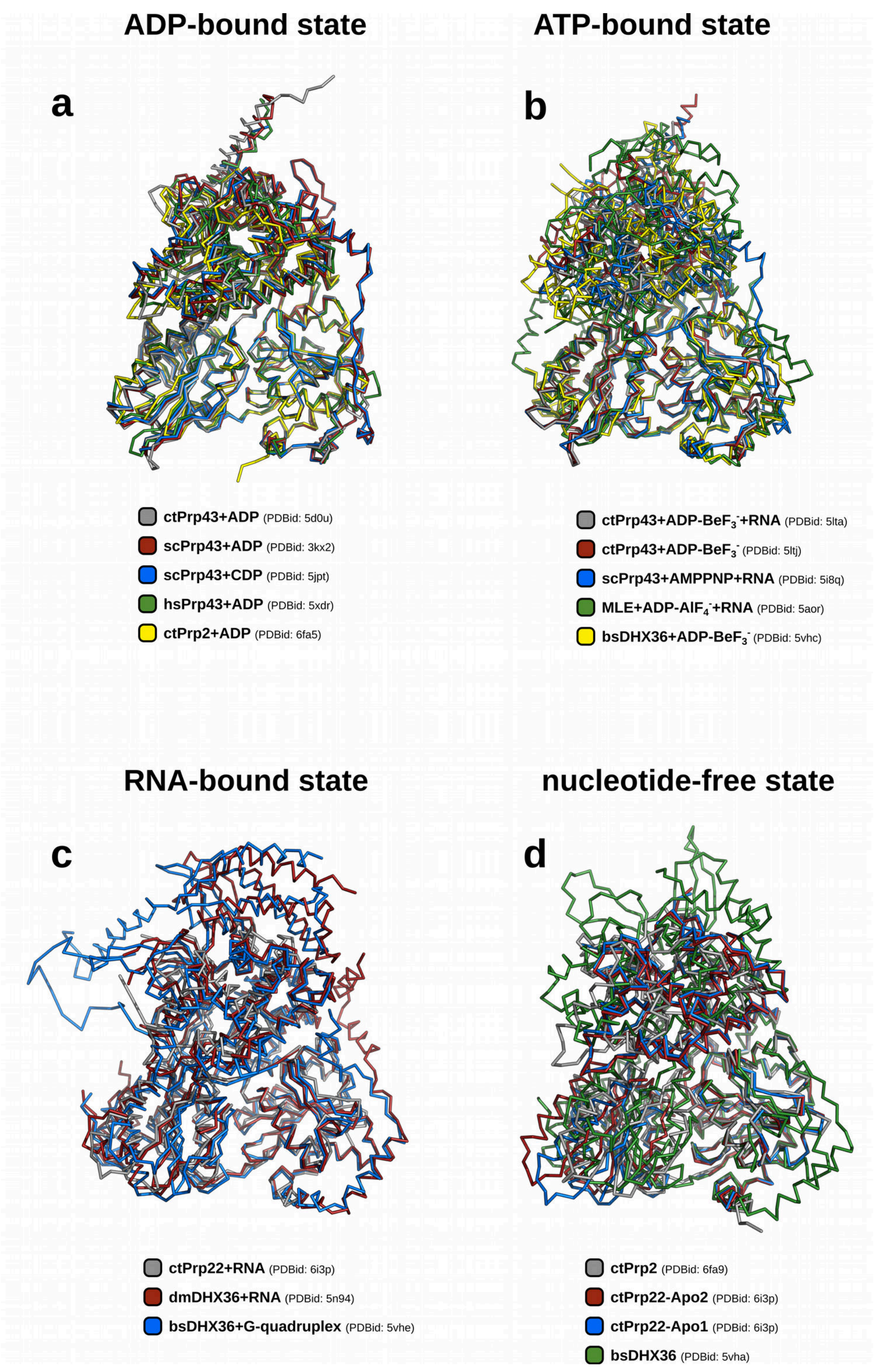

<u>**Supplemental figure S6**</u>

Structural comparison of different DExH-box ATPase-specific catalytic states. All structures are superimposed *via* the RecA1 domain. Structures with bound NDP (a), ATP-analog (b) and only RNA (c) exhibit a highly conserved helicase core conformation that is specific for each catalytic state. In contrast, adenosine nucleotide- and RNA-free structures (d) show divergent positions of the RecA2 domain.

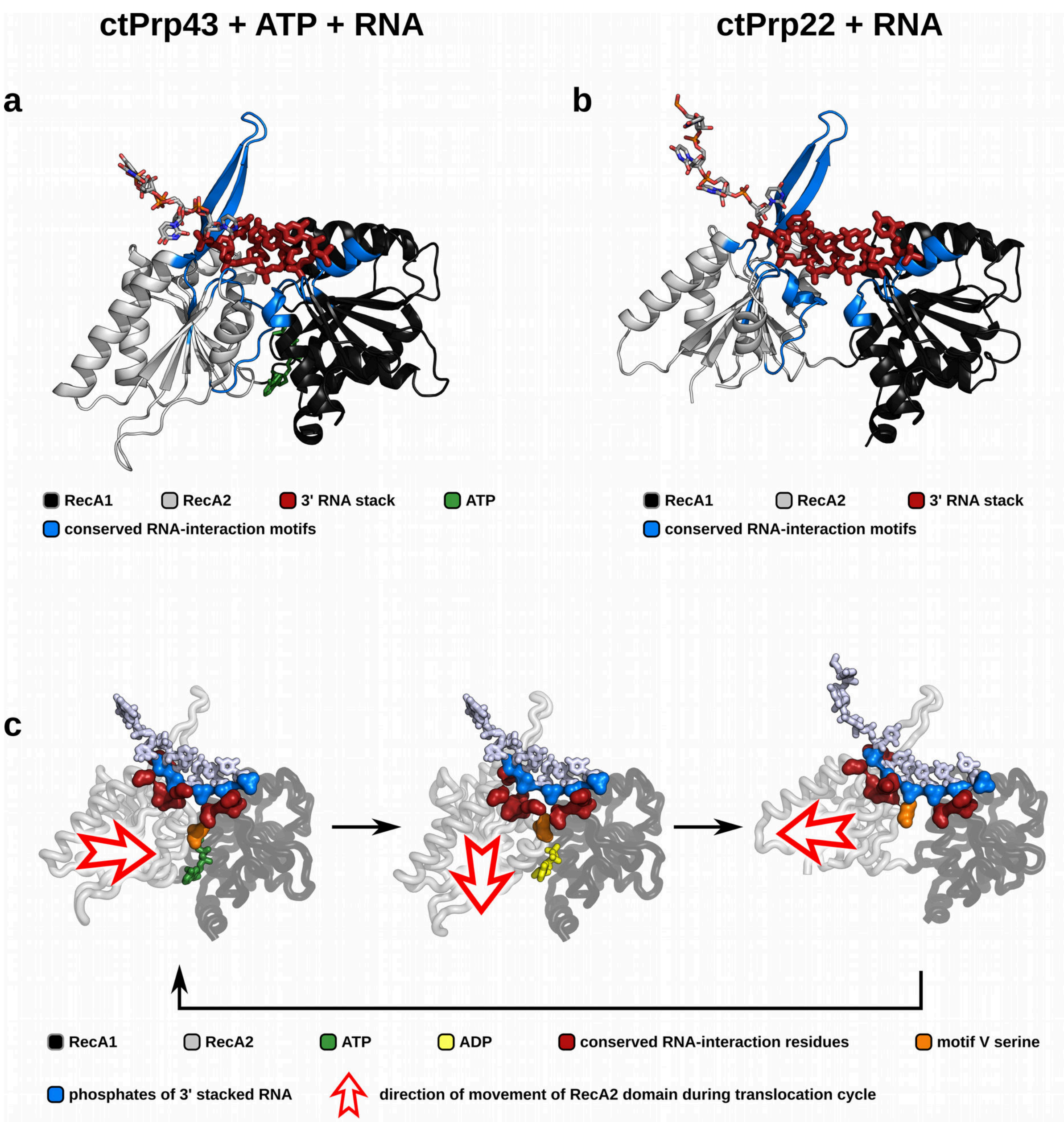

Supplemental figure S7

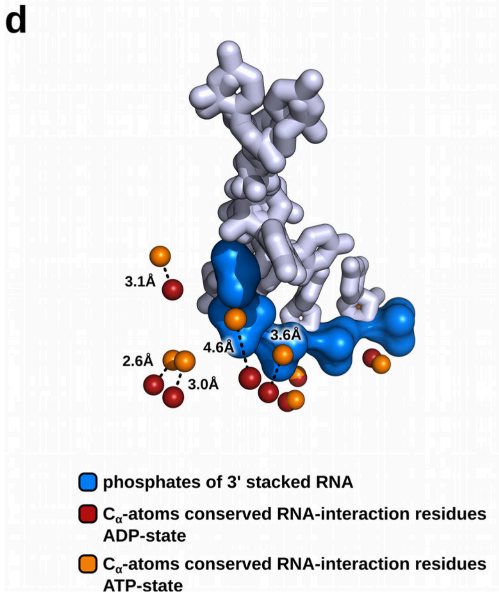

Structural dynamics of the helicase core during a translocation cycle. (a) The bound four-nucleotide stack (red) is maintained by interactions with conserved sequence motifs [motif Ia, Ib, IV and V] and structural elements [hook-turn, hook-loop and β-hairpin] (blue) in the closed ATP-bound state. The same interactions bind a five-nucleotide stack in the open adenosine nucleotide-free state (b). (c) The translocation dynamics depend on the movement of the RecA2 domain. ATP (green) hydrolysis induces a rotation of the RecA2 domain that interrupts the interactions of the RecA2 domain (red) with the phosphates (blue) of the four-nucleotide stack. Upon ADP (yellow) release the RecA2 domain gains an increased mobility which allows it to bind again to the RNA shifted by one nucleotide towards the 5' end. Closure of this open helicase core conformation is induced by the binding of ATP and the RNA is pushed through the tunnel towards 3' direction. (d) The rotation of the RecA2 domain induced by the transition from ATP to ADP, displaces the RNA-interaction residues of this domain by 2.6 – 4.6 Å. This interrupts the interaction of the RecA2 domain with the RNA-backbone when ADP is bound.

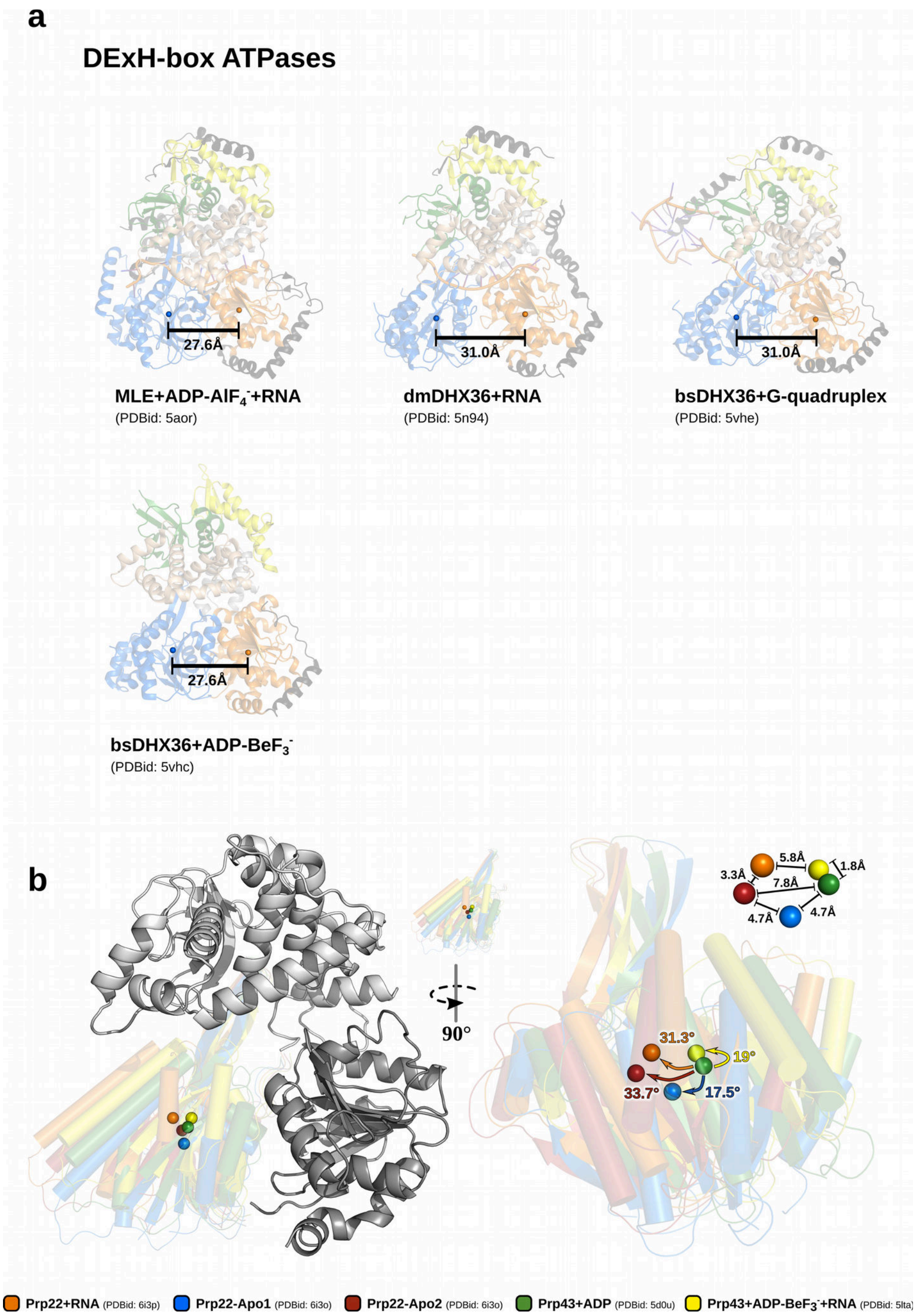

<u>Supplemental figure S8</u>

Overview of RecA1-RecA2 domain distances of DExH-box ATPases and RecA2 dynamics. (a) All structures are depicted as semi-transparent cartoon models and colored according to figure 1. Additional C-terminal domains of DExH-box ATPases are colored in yellow. Center of mass of the RecA-like domains are displayed as spheres and accordingly colored. In order to calculate the centers of mass for the same sets of atoms, the RecA1 and RecA2 domains of the ctPrp22-RNA complex were aligned with the corresponding domains of the individual catalytic states and center of mass for this superposed domains were determined. (b) RecA1 and C-terminal domains of ctPrp22 are depicted as cartoon models in different shades of gray. The RecA2 domains of different DEAH-box specific catalytic states are displayed in different colors (orange: RNA-bound, blue and red: Apo, green: ADP-bound, yellow: ATP- and RNA-bound). The position of the RecA2 domain relative to the RecA1 domain was achieved by superimposing the RecA1 domains of the individual catalytic states. The center of mass of each RecA2 domain is shown as an accordingly colored sphere. In order to calculate the center of mass for the same set of atoms, the RecA2 domain of the ctPrp22-RNA complex was aligned with the RecA2 domains of the individual catalytic states and center of mass for this superposed domain was determined. The angles of rotation as well as the translational shifts the RecA2 domain has to undergo are given.

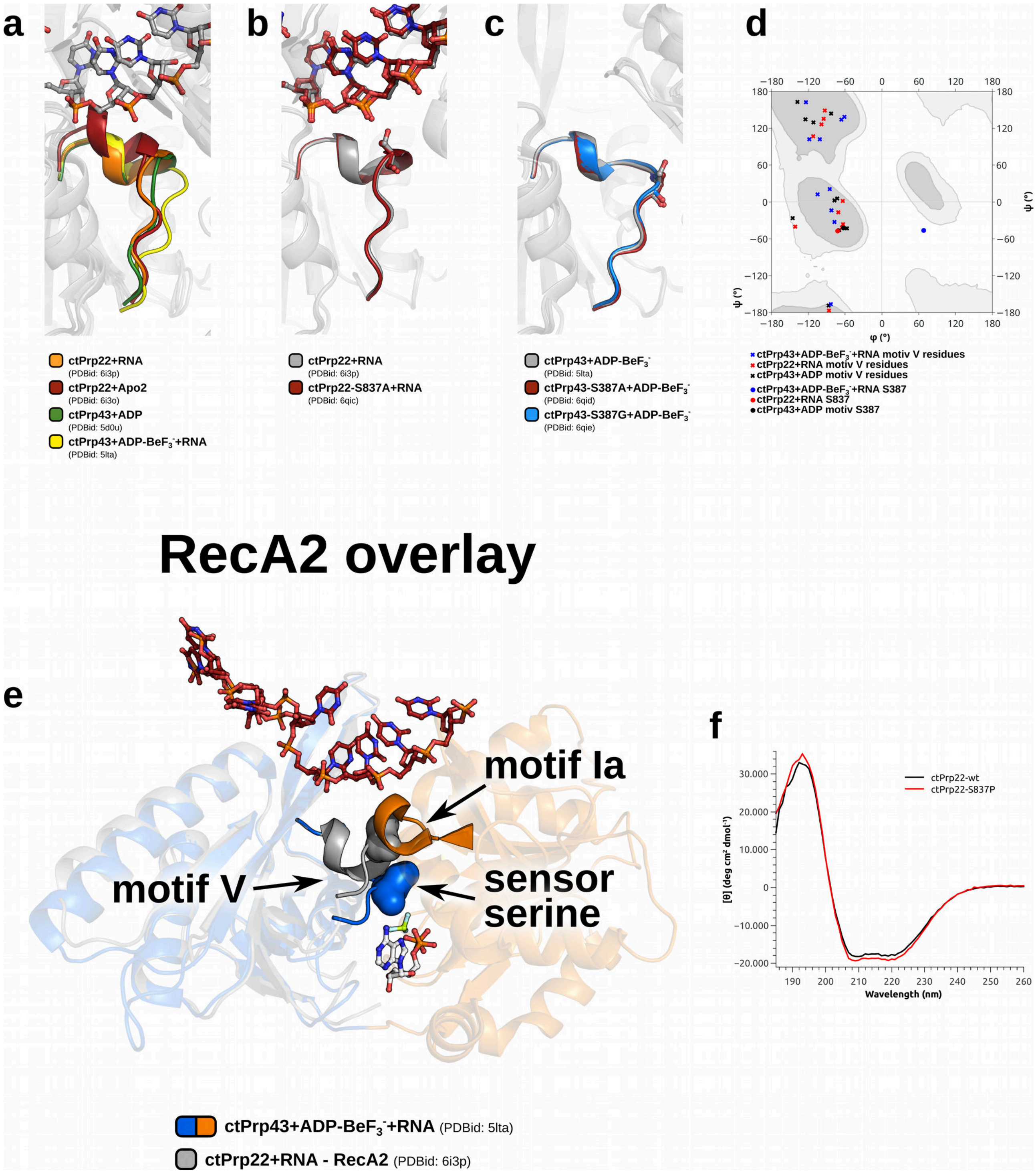

<u>**Supplemental figure S9**</u>

Motif V conformations. (a) The alignment of different catalytic states of genuine DEAH-box ATPases *via* the RecA2 domain highlights that motif V always adopts a mostly helical conformation with the exception of the ATP-bound state. There the α-helix is heavily distorted mainly due to an extensive interaction network of a serine with numerous components of the active site. Motifs V of ctPrp22-S837A (b), ctPrp43-S387A (c) and ctPrp43-S387G (c) adopt the same adenosine nucleotide-dependent conformations as the corresponding wildtype. (d) Ramachandran plot of motif V residues (crosses) of the different catalytic states. The motif V serine (dots) exhibits the most prominent difference as its interactions in the ATP-bound state forces its mainchain into an unfavored conformation. (e) An overlay of the ctPrp22 RecA2 domain of the RNA complex structure (grey) with the RecA2 domain of ctPrp43 in the ATP-bound state (blue) shows that the helical motif V would clash with motif Ia of the RecA1 domain (orange) in the ATP-bound state. The sensor serine is highlighted as a surface representation. (f) Circular dichroism spectra of ctPrp22 and ctPrp22-S837P are almost identical and confirm the structural integrity of the mutated protein.

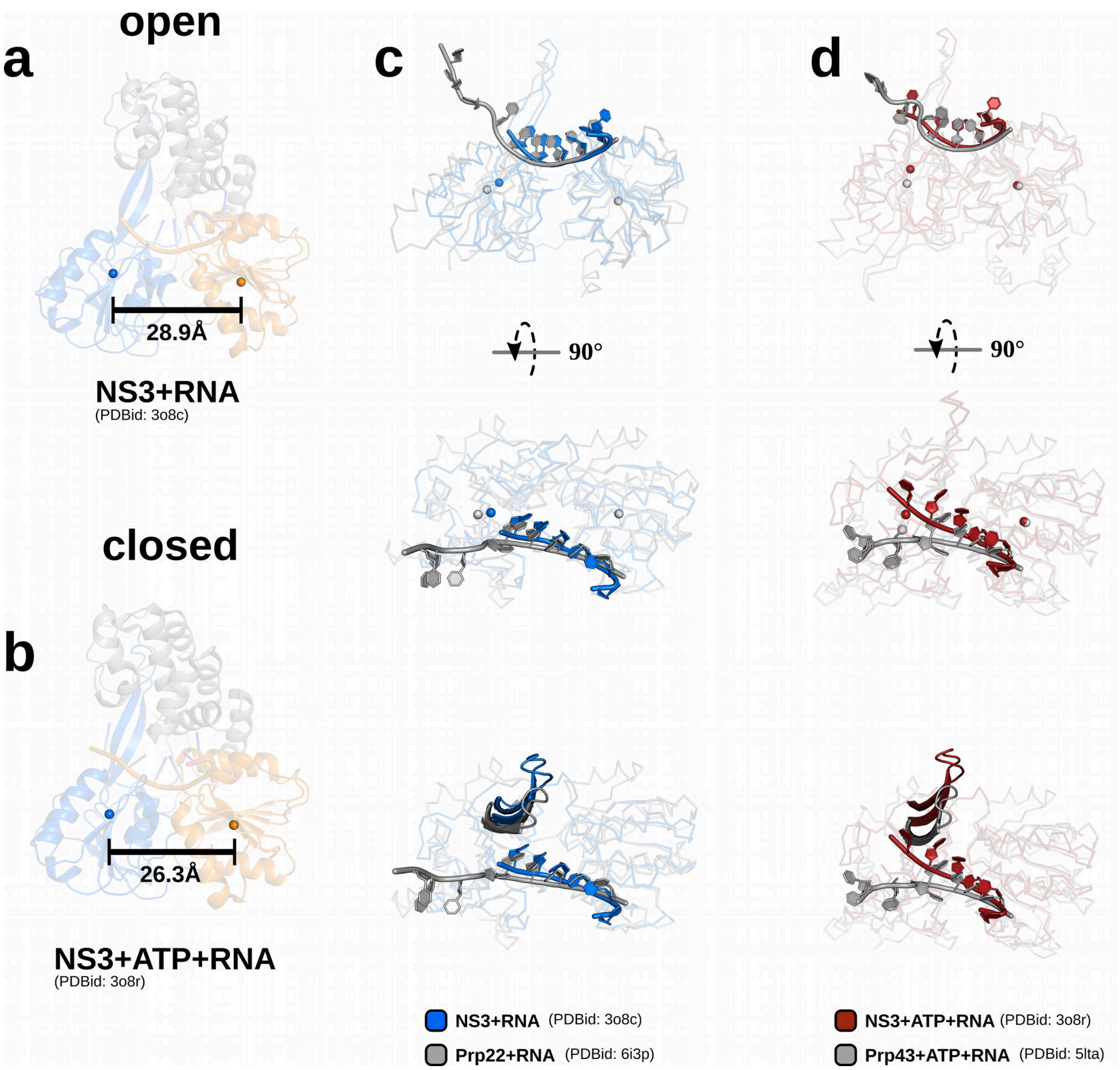

<u>**Supplemental figure S10**</u>

Helicase core conformation in NS3. (a) and (b) show RecA1-RecA2 center of mass distances in the RNA-bound and ATP- and RNA-bound states. HCV NS3 depiction and center of mass determination were performed as described in Fig. 3. The helicase core distance is slightly reduced in both states compared to the distances of DExH-box ATPases. A comparison of the RecA2 domain upon RecA1 domain superposition (c & d) reveals slightly different RecA2 positioning despite conserved open and close state conformations in DEAH-box ATPases and NS3. This divergent positioning of the RecA2 domain and different interactions with C-terminal domains leads to a different trajectory of the ssRNA through the binding tunnel.

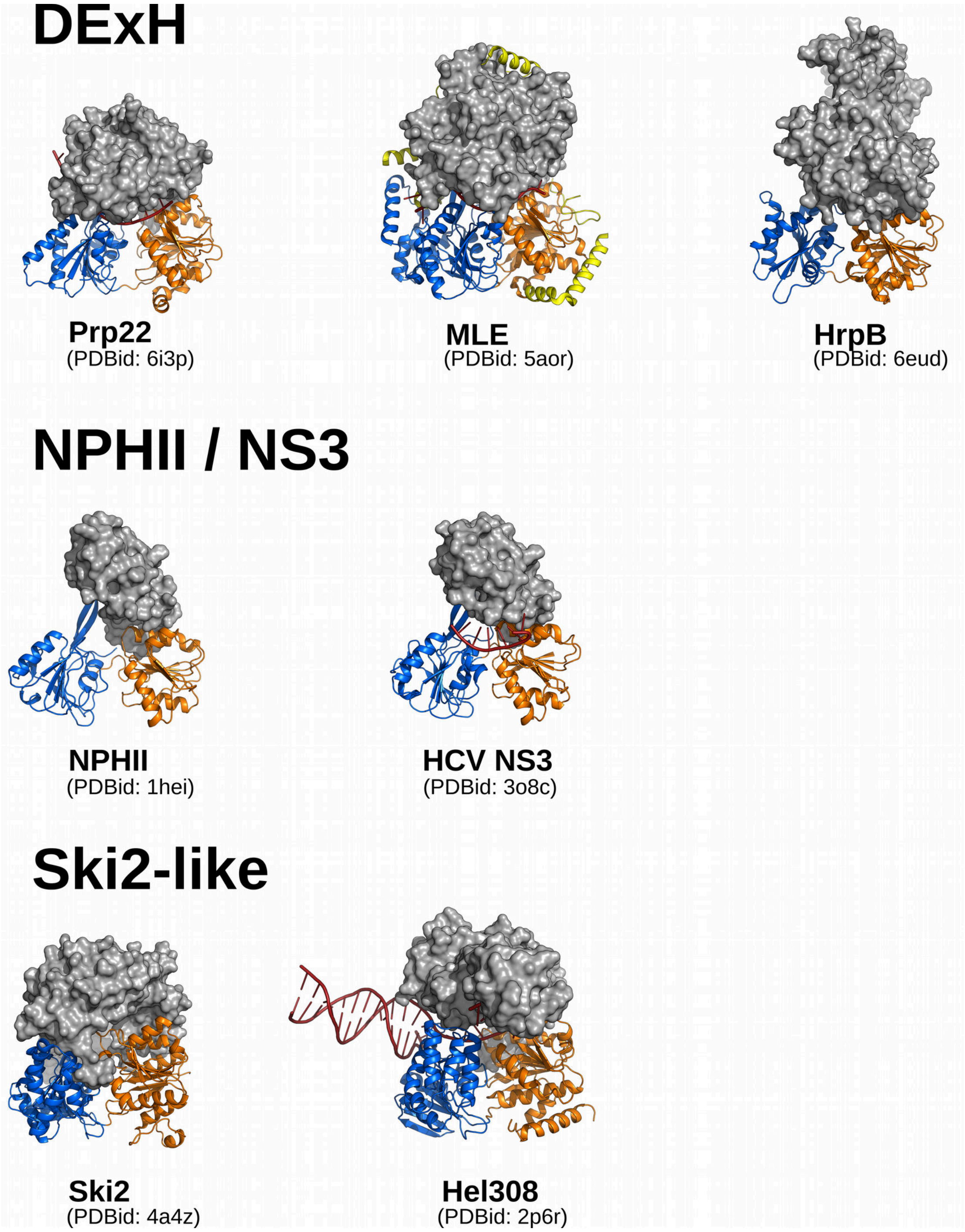

Supplemental figure S11

Overview of C-terminal domains of SF2 helicases. Although the helicase core is highly conserved in SF2 helicases, the C-terminal domains differ significantly in shape, size and composition of subdomains.

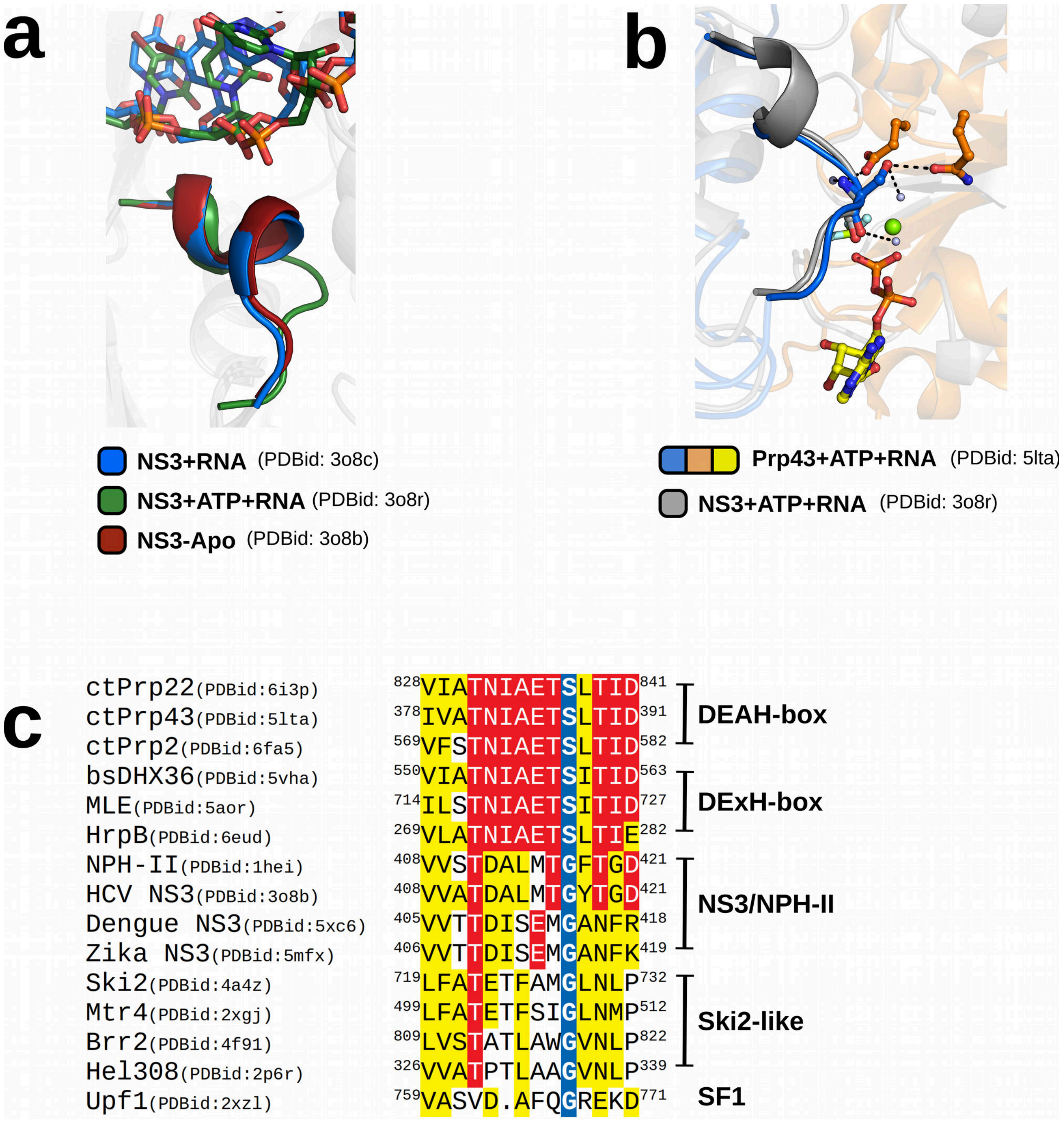

Supplemental figure S12

Motif V conservation. (a) NS3 motif V also adopts a relaxed helical conformation in adenosine nucleotide-free states and is distorted with bound ATP. (b) Although the motif V serine of DExH-box ATPases is not conserved in NS3 and replaced by a glycine, the overall conformation of the motif in the ATP-bound state is virtually identical. (c) Sequence alignment of motif V in different structurally known SF2 members. The motif V serine is strictly conserved in DExH-box ATPases but replaced by a conserved glycine in all other SF2 subfamilies.

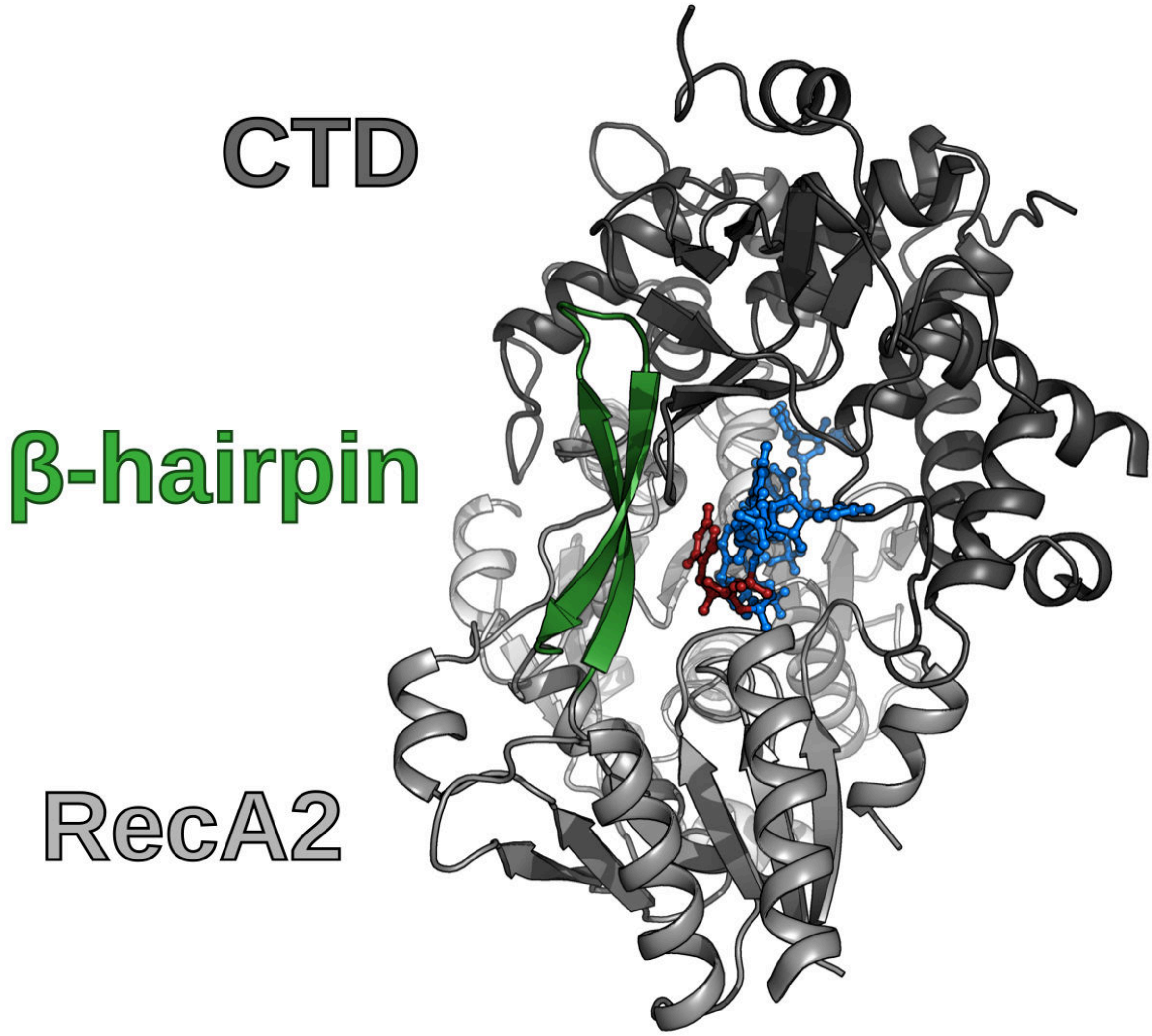

<u>**Supplemental movie SM1**</u>

Movement of RecA2 domain incorporates additional RNA-nucleotide in binding tunnel. The morphing of the transition from the ATP-bound ctPrp43 structure towards the adenosine nucleotide-free ctPrp22 structure highlights the movements of the RecA2 domain and its implications on the bound RNA. Due to the opening of the helicase core by the movement of the RecA2 domain, the β-hairpin (green) is also shifted and creates enough space for the incorporation the next 5’ RNA-nucleotide (red) between the hairpin and the four-nucleotide stack of the 3’ RNA region. This leads to the stack of five RNA-nucleotides in the adenosine nucleotide-free state.

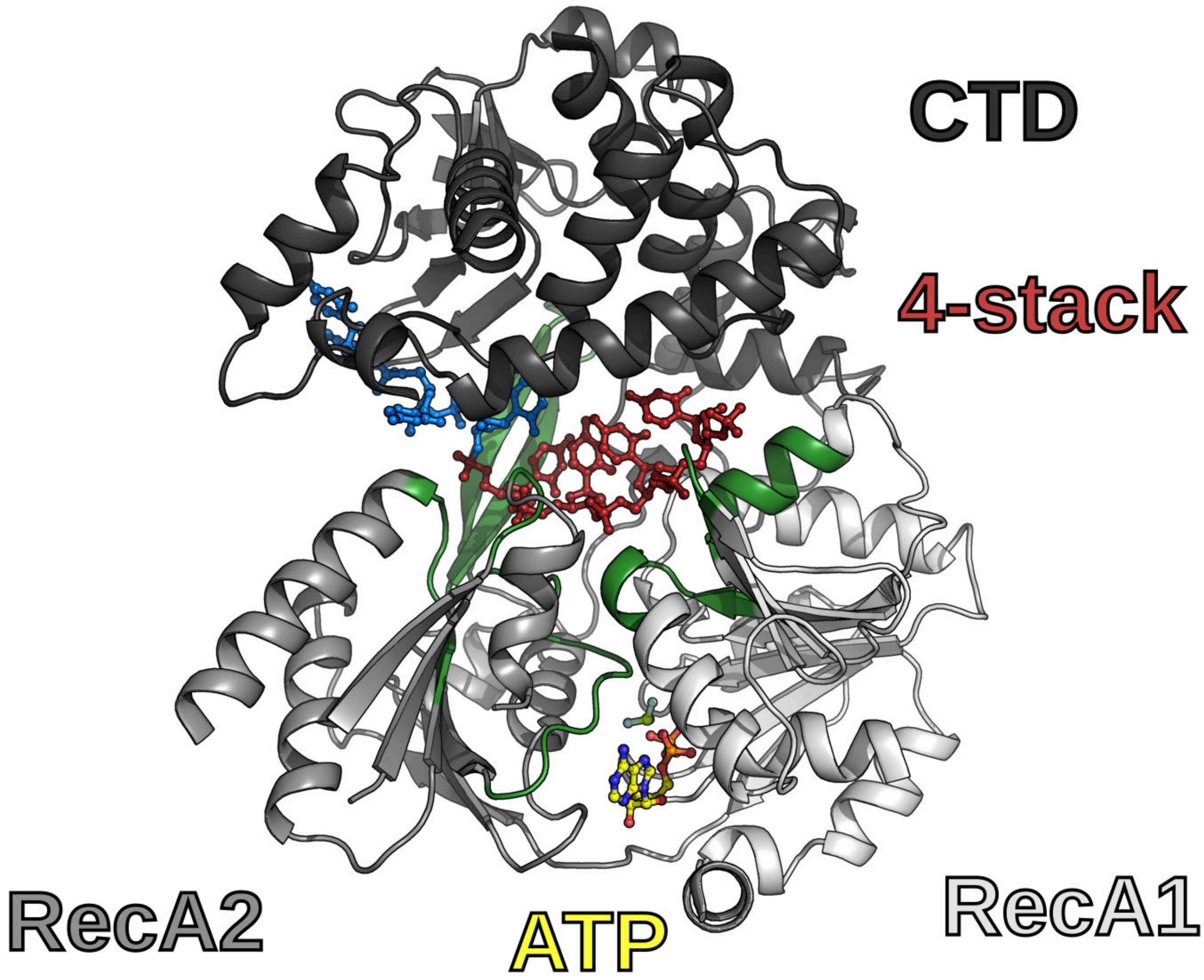

Supplemental movie SM2

RecA2 dynamics during one step of translocation. A morphing between the ATP-bound (ctPrp43; PDBid: 5lta), ADP-bound (ctPrp43; modeled using PDBid: 5d0u and PDBid: 5lta) and adenosine nucleotide-free (ctPrp22; PDBid: 6i3p) states illustrates the movements of the RecA2 domain needed for one step of translocation. In the ATP-bound state the conserved RNA-binding sequence and structural motifs (green) bind a stack of four RNA-nucleotides (red) in the binding tunnel. Upon hydrolysis of ATP, the RecA2 domain rotates by 19° and in the ADP-bound state the RNA-binding motifs of this domain loose the contact to the RNA. After the release of the ADP, the RecA2 domain shifts 6.5 Å towards the 5' end of the RNA and binds the RNA shifted by one nucleotide position. This allows the incorporation of one additional RNA-nucleotide in the binding tunnel leading to a bound five nucleotide stack (red). ATP-binding triggers closure of the helicase core and the RecA2 domain is shifted by 5.8 Å towards the RecA1 domain, pushing the RNA through the tunnel. Consecutive cycling of these events allows the DEAH-box ATPases to translocate along an ssRNA in 3'-5' direction.

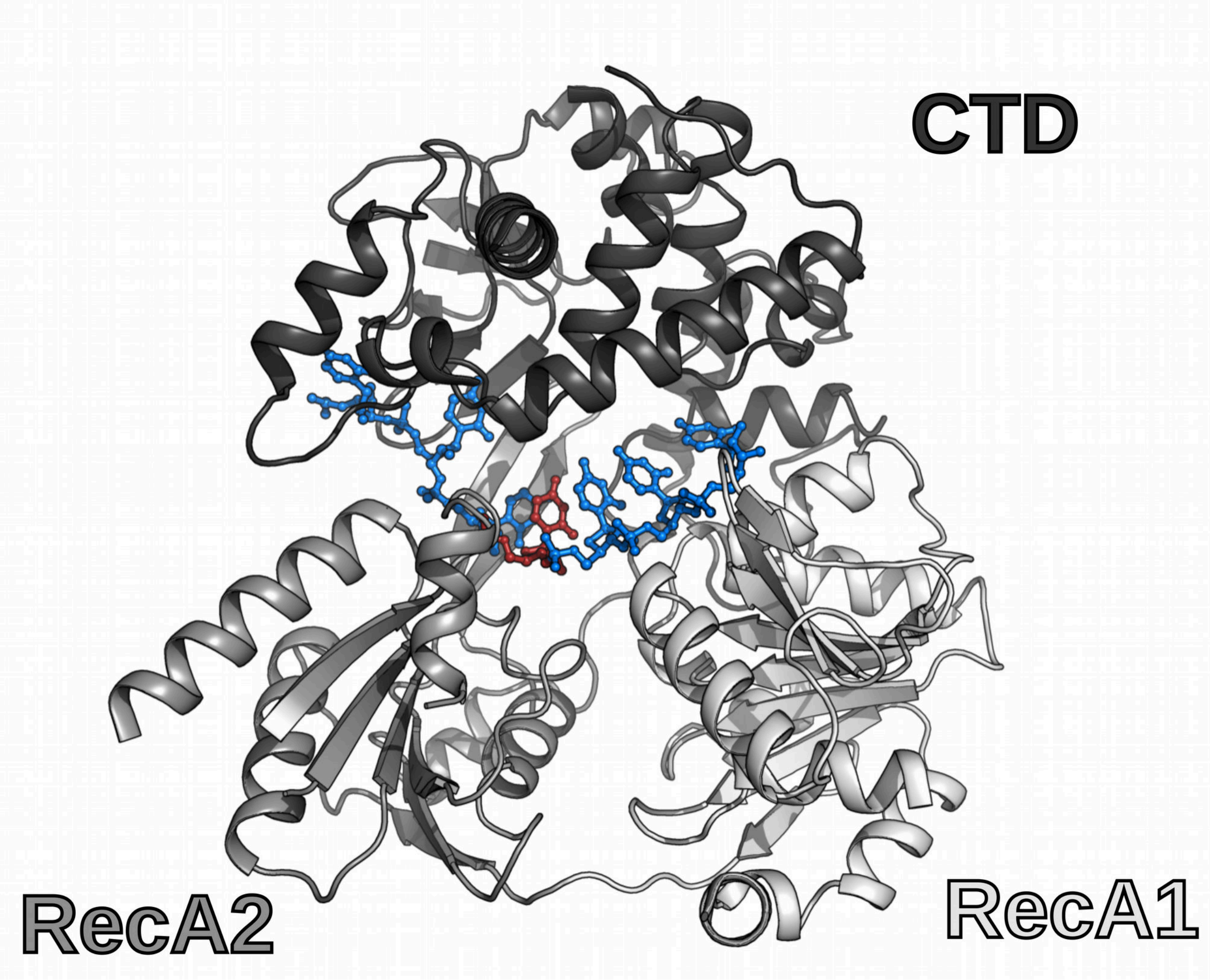

Supplemental movie SM3

DEAH-box ATPases translocate in 3'-5' direction at a step-size of one RNA-nucleotide per hydrolyzed ATP. Morphing of the transition between the closed ATP-bound conformation of ctPrp43 and the open adenosine nucleotide-free conformation of ctPrp22 in a continuous loop. The highlighted RNA-nucleotide (red) moves through the binding tunnel at a pace of one RNA-nucleotide per closed/open cycle, which represent one ATP hydrolysis cycle. This morphing envisions how DEAH-box ATPases translocate along an ssRNA by moving in 5' direction (3'-5' processivity) at a step-size of one RNA-nucleotide per hydrolyzed ATP.

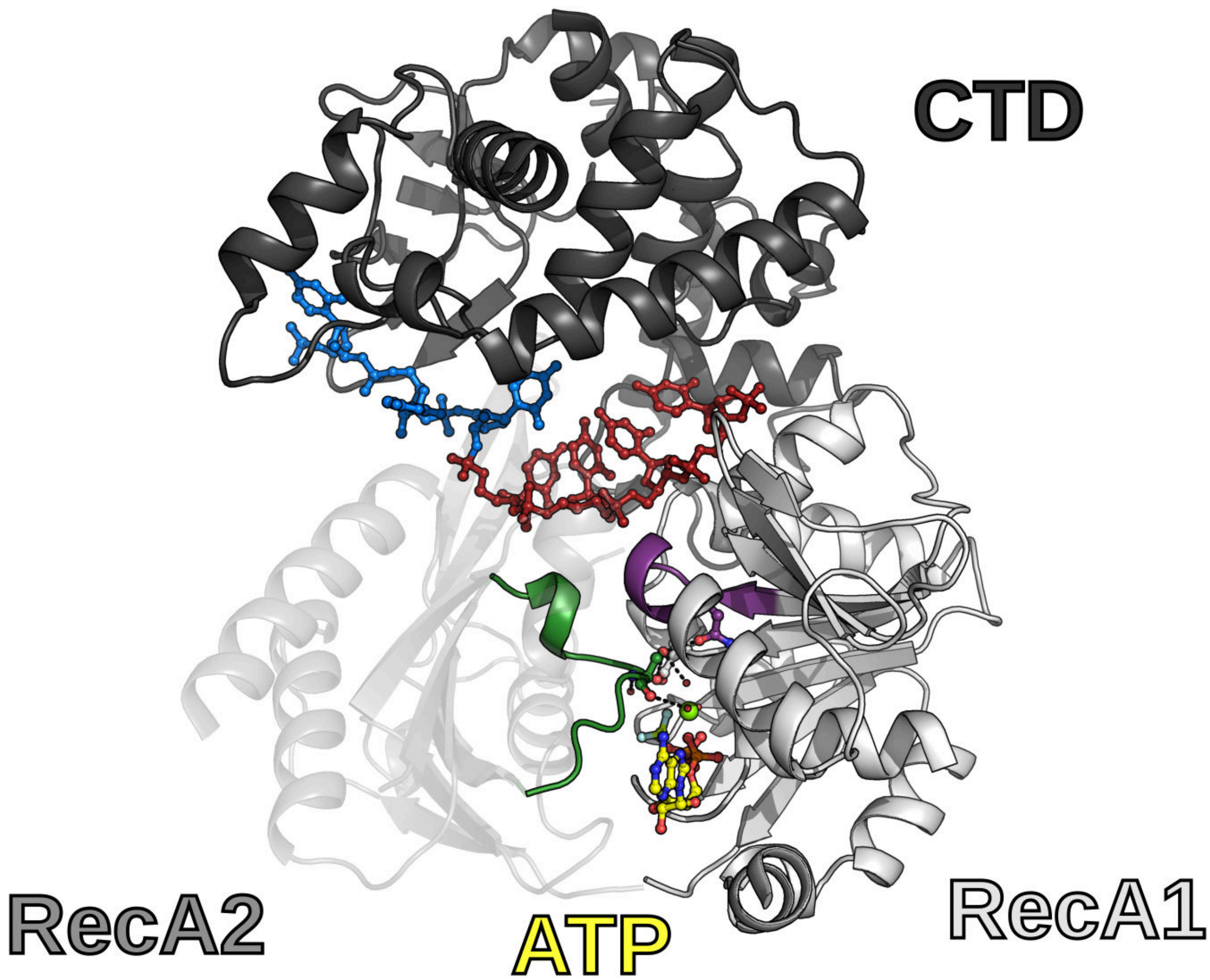

Supplemental movie SM4

Motif V senses catalytic state and switches between helical and distorted conformation. Motif V of DEAH-box ATPases (green) is located in the RecA2 domain and a serine in this motif (ball and stick model) senses the catalytic state by interaction with either the adenosine nucleotides or the ssRNA. Motif V adopts a helical conformation with bound ADP and in the absence of an adenosine nucleotide, which is distorted by the interactions of the serine with conserved active site components in the ATP-bound state. A helical conformation of motif V in the ATP-bound state would clash with motif Ia (purple) of the RecA1 domain.
